# Effect of a Mobile App on Prehospital Medication Errors During Simulated Pediatric Resuscitation: A Randomized Clinical Trial

**DOI:** 10.1001/jamanetworkopen.2021.23007

**Published:** 2021-08-30

**Authors:** Johan N. Siebert, Laurie Bloudeau, Christophe Combescure, Kevin Haddad, Florence Hugon, Laurent Suppan, Frédérique Rodieux, Christian Lovis, Alain Gervaix, Frédéric Ehrler, Sergio Manzano

**Affiliations:** 1 Department of Pediatric Emergency Medicine, Geneva Children’s Hospital, Geneva University Hospitals, Geneva, Switzerland; 2 Faculty of Medicine, University of Geneva, Geneva, Switzerland; 3 ACE Geneva Ambulances SA, Geneva, Switzerland; 4 Division of Clinical Epidemiology, Department of Health and Community Medicine, University of Geneva and Geneva University Hospital, Geneva, Switzerland; 5 Department of Emergency Medicine, Geneva University Hospitals, Geneva, Switzerland; 6 Division of Clinical Pharmacology and Toxicology, Department of Anesthesiology, Clinical Pharmacology, Intensive Care and Emergency Medicine, Geneva University Hospitals, Geneva, Switzerland; 7 Division of Medical Information Sciences, Department of Radiology and Medical Informatics, Geneva University Hospitals, Geneva, Switzerland

## Abstract

**Importance:**

Medication errors are a leading cause of injury and avoidable harm, affecting millions of people worldwide each year. Children are particularly susceptible to medication errors, but innovative interventions for the prevention of these errors in prehospital emergency care are lacking.

**Objective:**

To assess the efficacy of an evidence-based mobile app in reducing the occurrence of medication errors compared with conventional preparation methods during simulated pediatric out-of-hospital cardiac arrest scenarios.

**Design, Setting, and Participants:**

This nationwide, open-label, multicenter, randomized clinical trial was conducted at 14 emergency medical services centers in Switzerland from September 3, 2019, to January 21, 2020. The participants were 150 advanced paramedics with drug preparation autonomy. Each participant was exposed to a 20-minute, standardized, fully video-recorded, realistic pediatric out-of-hospital cardiac arrest cardiopulmonary resuscitation scenario concerning an 18-month-old child. Participants were tested on sequential preparations of 4 intravenous emergency drugs of varying degrees of preparation difficulty (epinephrine, midazolam, 10% dextrose, and sodium bicarbonate).

**Intervention:**

Participants were randomized (1:1 ratio) to the support of an app designed to assist with pediatric drug preparation (intervention; n = 74) or to follow conventional drug preparation methods without assistance (control; n = 76).

**Main Outcomes and Measures:**

The primary outcome was the rate of medication errors, defined as a failure in drug preparation according to predefined, expert consensus–based criteria. Logistic regression models with mixed effects were used to assess the effect of the app on binary outcomes. Secondary outcomes included times to drug preparation and delivery, assessed with linear regression models with mixed effects.

**Results:**

In total, 150 advanced paramedics (mean [SD] age, 35.6 [7.2] years; 101 men [67.3%]; mean [SD] time since paramedic certification, 8.0 [6.2] years) participated in the study and completed 600 drug preparations. Of 304 preparations delivered using the conventional method, 191 (62.8%; 95% CI, 57.1%-68.3%) were associated with medication errors compared with 17 of 296 preparations delivered using the app (5.7%; 95% CI, 3.4%-9.0%). When accounting for repeated measures, with the app, the proportion of medication errors decreased in absolute terms by 66.5% (95% CI, 32.6%-83.8%; *P* < .001), the mean time to drug preparation decreased by 40 seconds (95% CI, 23-57 seconds; *P* < .001), and the mean time to drug delivery decreased by 47 seconds (95% CI, 27-66 seconds; *P* < .001). The risk of medication errors varied across drugs with conventional methods (19.7%-100%) when compared with the app (4.1%-6.8%).

**Conclusions and Relevance:**

Compared with conventional methods, the use of a mobile app significantly decreased the rate of medication errors and time to drug delivery for emergency drug preparation in a prehospital setting. Dedicated mobile apps have the potential to improve medication safety and change practices in pediatric emergency medicine.

**Trial Registration:**

ClinicalTrials.gov Identifier: NCT03921346

## Key Points

**Question:**

Does the use of an evidence-based, custom-designed, mobile app result in decreased rates of pediatric medication errors compared with conventional preparation methods in prehospital emergency care?

**Findings:**

In this multicenter, simulation-based, randomized clinical trial including 150 advanced paramedics in 14 emergency medical services centers and 600 drug preparations, the proportion of medication errors committed during sequential preparation of 4 intravenous emergency drugs in prehospital settings was significantly decreased with the use of the app in absolute terms by 66.5%.

**Meaning:**

Dedicated mobile apps have the potential to change practices in prehospital emergency medicine and to improve quality of care in pediatric populations by decreasing the rate of medication errors.

## Teaser

This randomized clinical trial compares the efficacy of conventional drug preparation methods with a custom-designed mobile app that assists emergency medical personnel in drug preparations during simulated out-of-hospital cardiac arrest resuscitation in children.

## Introduction

Medication errors affect approximately 56 000 children treated by emergency medical services (EMS) each year in the US, with many drugs administered outside the proper dose range.^1^ In addition, many errors likely go underreported because of failure to recognize them or reluctance to report them.^2^ In 2017, the World Health Organization called for a reduction by 50% of serious and avoidable medication-associated harm in all countries during the ensuing 5 years.^3^ Emergency care is an environment with a high risk for medication errors, particularly in critical pediatric situations, such as out-of-hospital cardiopulmonary resuscitation.^4^ In this setting, the combination of limited safeguards and resources^5^ places children at higher risk than adults for life-threatening prehospital medication errors.^5,6^ Factors associated with increased risk for children include little exposure of paramedics to critically ill children, an increased cognitive load owing to emotional stress and time pressure, and pediatric-specific, age-related variations in pharmacokinetics, with the need for an individual, weight-based dose calculation and drug preparation for each child.^1,7,8^ Among other drugs, epinephrine has the highest rate of incorrect dose administration, with up to 68% of preparations containing an error and a mean error overdose of 808%.^9,10^ Similarly, a study^11^ indicated a frequency of medication errors by paramedics of 49% to 63%, with miscalculation as a primary cause.

Although numerous interventions involving information technology have been developed to improve in-hospital security of the medication process,^12^ error prevention strategies and evaluation of their efficacy in the prehospital area are scarce.^1,13^ Mobile device apps are increasingly used to improve health care quality and safety performance. However, most of the few available apps within the field of cardiopulmonary resuscitation are of marginal medical value and have limited usability and poor user friendliness.^14^ Whether these apps actually improve or impede clinical care is unknown, especially in pediatrics, because the efficacy of most of the apps has not been well validated.^14^ Thus, the need for requirement-driven digital health solutions development and systematic validation with clinical studies has become increasingly essential.^15^

Previous trials^16,17^ have reported the ability of a medical app, PedAMINES (Pediatric Accurate Medication in Emergency Situations), to significantly decrease in-hospital medication error rates for continuous infusions and time to drug delivery compared with conventional preparation methods during simulation-based pediatric resuscitations. Although similarities exist, the prehospital environment is distinctly different in many aspects. We designed this study to evaluate the efficacy of PedAMINES to decrease pediatric medication errors by facilitating the preparation of drugs for intravenous administration during pediatric out-of-hospital cardiac arrest.

## Methods

### Design

This open-label, simulation-based, multicenter, randomized clinical trial was conducted at 14 urban EMS centers in Switzerland covering a population of more than 2.3 million people from September 3, 2019, to January 21, 2020. The trial protocol has been published^18^ and is given in Supplement 1. The trial was approved and received a declaration of no objection (waiver) by the Geneva Cantonal Ethics Committee/SwissEthics, Switzerland. All participants provided written informed consent that was obtained in a manner consistent with the Declaration of Helsinki.^19^ No one received compensation or was offered any incentive for participating in this study. The trial was performed in accordance with appropriate guidelines^20,21^ and followed the Consolidated Standards of Reporting Trials (CONSORT) reporting guideline.^22^

We evaluated 2 different methods to guide the preparation of emergency drugs for direct intravenous administration at pediatric doses during a standardized, simulated, pediatric out-of-hospital cardiac arrest using a high-fidelity manikin (full details of the setting and scenario are provided in the eMethods in Supplement 2). Participants were randomly assigned to prepare the drugs either with the support of the app (intervention group) or by conventional methods (control group).

### Participants

Eligible participants were registered advanced paramedics working in EMS who had undergone a 3-year formal educational program in Switzerland, a pluralistic country with 4 official languages without uniformly standardized or benchmarked EMS clinical guidelines, protocols, or operating procedures, similar to many countries, including the US. During their education, paramedics were trained in advanced life support procedures, including defibrillation, airway management, peripheral intravenous line cannulation, and the administration of medications to ensure advanced and independent emergency prehospital care. The study excluded emergency medical technicians because they have no drug preparation autonomy.

### Randomization

Randomization with a 1:1 ratio was stratified by EMS center. Random block sizes were used to generate the randomization lists by means of web-based software.^23^ Concealment of the randomized assignment was ensured with the allocation software and was not released until participants started the scenario. Participants were unaware of the scenario and drugs intended for use during recruitment to minimize preparation bias.

### Intervention

**Figure 1.**
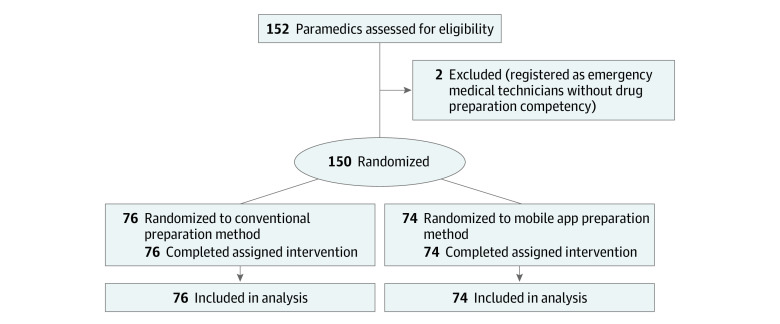
CONSORT Diagram of Study Participation

On the day of participation after randomized allocation, each participating paramedic was required to (1) complete a survey collecting data regarding their demographic characteristics, health care training, and simulation and computer experience; (2) attend a standardized 5-minute training session on how to use the mobile app (thus providing identical preliminary education); and (3) attend a presentation of the simulation manikin characteristics. Each participant was then exposed to a 20-minute, standardized, fully video-recorded, highly realistic pediatric out-of-hospital cardiac arrest cardiopulmonary resuscitation scenario concerning an 18-month-old child. They were asked to sequentially prepare and intravenously inject 4 different drugs of varying degrees of preparation difficulty (epinephrine [0.01 mg/kg], midazolam [0.1 mg/kg], 10% dextrose [4 mL/kg], and sodium bicarbonate [1 mmol/kg]) with the support of the app designed to assist with pediatric drug preparation (eFigure 1 in Supplement 2)^24^ or by following conventional pediatric drug preparation methods (ie, without app support) (Figure 1). The rationale for the selection of these drugs and the required preparation steps are provided in the eMethods in Supplement 2. The weight (12 kg) of the child (manikin) was told to each participant at the beginning of the resuscitation scenario by 1 of us (S.M.) and iteratively repeated with each new drug preparation. Full details of the scenario and data collection are provided in the eMethods in Supplement 2.

### Outcomes

The primary outcome was medication error, defined as a failure in drug preparation if at least 1 of the following errors was committed: a deviation in drug dose higher than 10% from the correct weight dose^25^; inability to calculate drug dose without guidance from the paramedic investigator (L.B.) leading the resuscitation in the room; or (owing to its clinical relevance) a deviation of higher than 10% of the final administered concentration of sodium bicarbonate from the prescribed 4.2% concentration.^26^ A dose deviation set margin was defined as a threshold set between 0% and 100% above and below which a drug dose deviated from the prescribed dose and was then considered an overdose or an underdose, respectively. The final correct volume of drugs to be drawn was not released to the paramedics. Although the intervention could not be masked, all investigators remained unaware of the outcomes until all data were unlocked for analysis at the end of the trial.

All videos were reviewed by 1 of us (J.N.S., a senior pediatric emergency physician and American Heart Association–certified Pediatric Advanced Life Support instructor). To assess the reproducibility of the video review procedure, another 1 of us (L.B., a senior advanced paramedic with Pre-Hospital Pediatric Life Support and Pediatric Advanced Life Support certifications) independently duplicated the review in a randomly selected 10% of all videos. Because blinding of the videos was not possible, both reviewers were not blinded to group assignment and the study hypothesis, but they were blinded to each other’s reviews. Study-specific training and standardization of the reviewers were ensured through their involvement in previous in-hospital studies^16,17^ and by their following of the predefined scenario. Secondary outcomes were the elapsed time in seconds between the oral prescription by the physician and time to both drug preparation completion and delivery by the participant.

### Statistical Analysis

The trial was designed with a 2-sided α = .05 and power of 90% to detect an absolute difference of at least 30% in proportions of medication errors between study groups (60% with the conventional method vs 30% with the app^9^), which was considered to be a sufficient difference to modify clinical practice. We needed 56 participants per study group, and we planned to recruit 60 paramedics per study group. Additional information regarding the sample size calculation has been published previously.^18^

Logistic (respectively linear) regression models with mixed effects were used to assess the effect of the app on binary (respectively continuous) outcomes. A random intercept was introduced in the models with EMS centers as a random effect. When the 4 drugs were analyzed simultaneously, 2 crossed random effects were added (participants and drugs). Odds ratios from logistic models were reported, and risk differences were obtained with a parametric bootstrap approach. When a logistic model could not be used owing to lack of outcome, the risk difference was assessed using the Miettinen-Nurminen approach^27^ to account for the stratified randomization. Prespecified subgroup analyses (ie, total number of emergency calls per year per EMS and paramedic experience expressed as years since certification) were performed by introducing an interaction term into the regression models. Dose deviations were investigated as follows: for each drug, the frequencies of underdoses and overdoses were assessed, including the median (interquartile range) relative dose deviations. The cumulative distribution of the absolute value of the relative dose deviation was graphically represented. The distribution of the relative dose deviation was compared between study groups by using the van Elteren test stratified by EMS center as initially planned, but *P* values are not reported because this was a secondary analysis.

Interrater reliability scores from video reviews were calculated using the Cohen κ coefficient for medication errors (eTable 1 in Supplement 2). Because the other outcomes were continuous variables, the Bland-Altman method was used to plot the difference of values reported by both reviewers against the mean value for each outcome (eFigure 2 in Supplement 2). The limits of agreement were assessed by an interval of plus or minus 1.96 SDs of the measurement differences on either side of the mean difference. The null hypothesis that there was no difference in the means between both reviewers was tested using a *t* test. Mean differences are reported with 95% CIs. In addition, the intraclass correlation coefficients for time to drug preparation and time to drug delivery were assessed assuming that raters were a sample from a larger population of possible raters. The agreement was investigated for the data on each drug.

All statistical tests were 2-sided with a 5% significance level. No correction for multiplicity was applied, and the 95% CI was not adjusted for multiplicity of analysis. Analyses were performed using R, version 4.0.2 (R Project for Statistical Computing). The R package lme4,^28^ version 1.1-26, was used to fit models with random effects, and the R package lmerTest,^29^ version 3.1-3, was used to obtain the *P* values for the fixed effects. The risk difference stratified by EMS center (assessed with the Miettinen-Nurminen approach) was evaluated with the R package ratesci,^30^ version 0.3-0.

## Results

A total of 150 advanced paramedics (mean [SD] age, 35.6 [7.2] years; 101 men [67.3%]; mean [SD] time since paramedic certification, 8.0 [6.2] years) underwent randomization to the 2 study groups. In total, 74 were assigned to the mobile app group and 76 to the conventional method, with no dropout or missing data (Figure 1). Baseline characteristics for the participants in the 2 groups are given in Table 1, and recruitment was balanced across EMS centers. Assessment of outcomes showed an excellent interrater agreement for the primary outcome, with a κ coefficient of 1 for all drugs (eTable 1 in Supplement 2). The intraclass correlation coefficient representing interrater reliability for the secondary outcomes was 1 (eFigure 2 in Supplement 2).

**Table 1.** Baseline Characteristics of Participants

Characteristic	Participants^a^	
	Mobile app (n = 74)	Conventional method (n = 76)
Age, mean (SD) [range], y	35.7 (7.3) [23-53]	35.5 (7.1) [22-53]
Sex		
Female	26 (35.1)	23 (30.3)
Male	48 (64.9)	53 (69.7)
Proficiency in the use of smartphones or tablets		
Strongly disagree	1 (1.4)	0
Disagree	3 (4.1)	3 (3.9)
Neutral	12 (16.2)	13 (17.1)
Agree	38 (51.4)	48 (63.2)
Strongly agree	20 (27.0)	12 (15.8)
Time since paramedic certification, y		
Mean (SD)	7.9 (6.2)	8.2 (6.3)
<5	26 (35.1)	27 (35.5)
5 to 10	29 (39.2)	26 (34.2)
>10	19 (25.7)	23 (30.3)
Specific pediatric training^b^		
Yes	37 (50.0)	35 (46.1)
No	37 (50.0)	41 (53.9)
Previous experience with simulation		
Yes	44 (59.5)	56 (73.7)
No	30 (40.5)	20 (26.3)
Time since last pediatric cardiopulmonary resuscitation, mo		
Never	32 (43.2)	30 (39.5)
≥24	26 (35.1)	28 (36.8)
12 to <24	11 (14.9)	11 (14.5)
6 to <12	4 (5.4)	5 (6.6)
<6	1 (1.4)	2 (2.6)
Time since last preparation of emergency drugs, mo		
Never	9 (12.3)	12 (15.8)
≥24	18 (24.7)	18 (23.7)
12 to <24	15 (20.5)	17 (22.4)
6 to <12	16 (21.9)	7 (9.2)
<6	15 (20.5)	22 (28.9)
Satisfaction with current drug preparation methods		
Very unsatisfied	9 (12.3)	8 (10.5)
Unsatisfied	18 (24.7)	19 (25.0)
Neutral	25 (34.2)	22 (28.9)
Satisfied	20 (27.4)	24 (31.6)
Very satisfied	1 (1.4)	3 (3.9)
Proficient with intravenous drug preparation		
Strongly disagree	9 (12.3)	7 (9.2)
Disagree	15 (20.5)	30 (39.5)
Neutral	30 (41.1)	14 (18.4)
Agree	18 (24.7)	22 (28.9)
Strongly agree	1 (1.4)	3 (3.9)
Attitude toward new technology		
Strongly unfavorable	0	0
Unfavorable	0	1 (1.3)
Neutral	6 (8.2)	1 (1.3)
Favorable	23 (31.5)	17 (22.4)
Strongly favorable	44 (60.3)	57 (75.0)

^a^ Data are presented as number (percentage) of participants unless otherwise indicated. Percentages may not total 100 because of rounding.

^b^ Includes Pediatric Advanced Life Support and Pre-Hospital Paediatric Life Support training.

### Primary Outcome

A total of 600 drug doses were delivered; 191 of 304 doses given using the conventional method (62.8%; 95% CI, 57.1%-68.3%) and 17 of 296 doses given using the mobile app (5.7%; 95% CI, 3.4%-9.0%) were associated with medication errors (Table 2). Most medication errors in drug preparations were attributable to a dose deviation higher than 10% of the prescribed dose (172 of 304 [56.6%] using the conventional method and 16 of 296 [5.4%] using the mobile app). Overall, when accounting for repeated measures, the risk of incorrect preparation of the 4 drugs was decreased by 66.5% (95% CI, 32.6%-83.8%; *P* < .001) using the mobile app. The difference remained significant between study groups even when setting higher dose deviation incremental margins up to 50% (eTable 2 in Supplement 2). The risk varied across drugs when using the conventional method, ranging from 19.7% for the third drug (10% dextrose) to 100% for the fourth drug (sodium bicarbonate), whereas it was approximately 5% for any drug when using the app (Table 2).

**Table 2.** Number and Proportion of Medication Errors

Variable	Medication errors, No./total No. (%)		Odds ratio (95% CI)^a^	Risk difference (95% CI)^b^	*P* value
	Mobile app (n = 296)	Conventional method (n = 304)			
Incorrect preparation	17/296 (5.7)	191/304 (62.8)	102.9 (38.9-271.1)	66.5 (32.6-83.8)	<.001
Dose deviation >10%	16/296 (5.4)	172/304 (56.6)	44.5 (20.2-97.8)	54.7 (29.9-72.9)	<.001
Assistance required	0/296	55/304 (18.1)	NA	18.1 (1.7-22.1)	<.001
Fourth drug concentration deviation >10%^c^	5/74 (6.8)	46/76 (60.5)	21.2 (7.6-58.6)	53.8 (39.8-64.9)	<.001
Incorrect preparation per drug					
First drug: epinephrine	4/74 (5.4)	44/76 (57.9)	27.9 (8.7-89.9)	53.8 (38.4-66.4)	<.001
Second drug: midazolam	5/74 (6.8)	56/76 (73.7)	38.6 (13.6-109.5)	66.9 (53.2-76.6)	<.001
Third drug: 10% dextrose	3/74 (4.1)	15/76 (19.7)	6.2 (1.7-22.8)	14.2 (4.5-27.2)	.007
Fourth drug: sodium bicarbonate	5/74 (6.8)	76/76 (100)	NA	93.2 (84.9-97.1)	<.001

Abbreviation: NA, not applicable.

^a^ Logistic regression models with mixed effects were used to assess the odds ratio accounting for the randomization stratified by emergency medical services centers.

^b^ The reported risk difference was obtained from the estimates of the models by using a parametric bootstrap. When a model did not converge because of the lack of outcome, only the risk difference stratified by centers was assessed and reported.^27^

^c^ Concentrations of 8.4% sodium bicarbonate (ie, the concentration provided to participants) should be administered through a peripheral intravenous line after first diluting by drawing up the required dose with the same volume of diluent to make a 4.2% final sodium bicarbonate solution to avoid thrombophlebitis and consecutive tissue damage caused by extravasation.^26^

All 76 participants (100%) using the conventional preparation method committed at least 1 preparation error during the whole scenario compared with 14 participants (18.9%) using the app. The proportion of participants committing an incorrect preparation was therefore decreased by 81.1% (95% CI, 70.6%-89.7%; *P* < .001) using the app (Table 3). Moreover, the proportion of participants committing several preparation errors (ie, at least for ≥2 drugs) was substantially lower with the app (4.1%) than with the conventional method (85.5%) (Table 3).

**Table 3.** Preparation Errors per Participant

Incorrect preparations per participant	Participants, No. (%)^a^	
	Mobile app (n = 74)	Conventional method (n = 76)
0	60 (81.1)	0
1	11 (14.9)	11 (14.5)
2	3 (4.1)^b^	27 (35.5)^c^
3	0^b^	26 (34.2)^c^
4	0^b^	12 (15.8)^c^

^a^ *P* < .001 overall.

^b^ Sum of 2, 3, and 4 incorrect preparations per participant is 4.1%.

^c^ Sum of 2, 3, and 4 incorrect preparations per participant is 85.5%.

Of the 172 preparations with a dose deviation in the control group, 42 (24.4%) were overdoses (median, 63% [range, 13%-1150%] of the prescribed dose), whereas 130 (75.6%) were underdoses (median, 58% [range, 11%-100%] of the prescribed dose) (eTable 3 in Supplement 2). In 39 preparations, a dose deviation was committed and assistance was required. For the fourth drug (sodium bicarbonate), the most complicated drug to prepare, the dilution step was more prone to errors. Of 76 preparations, 36 (47.4%) contained the drug only without dilution, of which 24 were below the target value. Of the remaining 40 preparations, 10 (13.2%) contained a final concentration deviating by more than 10% from the prescribed dose, 23 (30.3%) were underdoses (median, 50% [range, 17%-98%] of the prescribed dose), and 7 (9.2%) required substantial assistance for preparation.

Of the 16 preparations with a dose deviation in the intervention group, 8 (50.0%) were overdoses (median, 175% [range, 11%-900%] of the prescribed dose and 8 (50.0%) were underdoses (median, 23% [range, 17%-100%] of the prescribed dose) (eTable 3 in Supplement 2). In the intervention group, no preparation required assistance from the paramedic investigator. Details regarding the medication errors committed with the app are shown in eTable 4 in Supplement 2.

**Figure 2.**
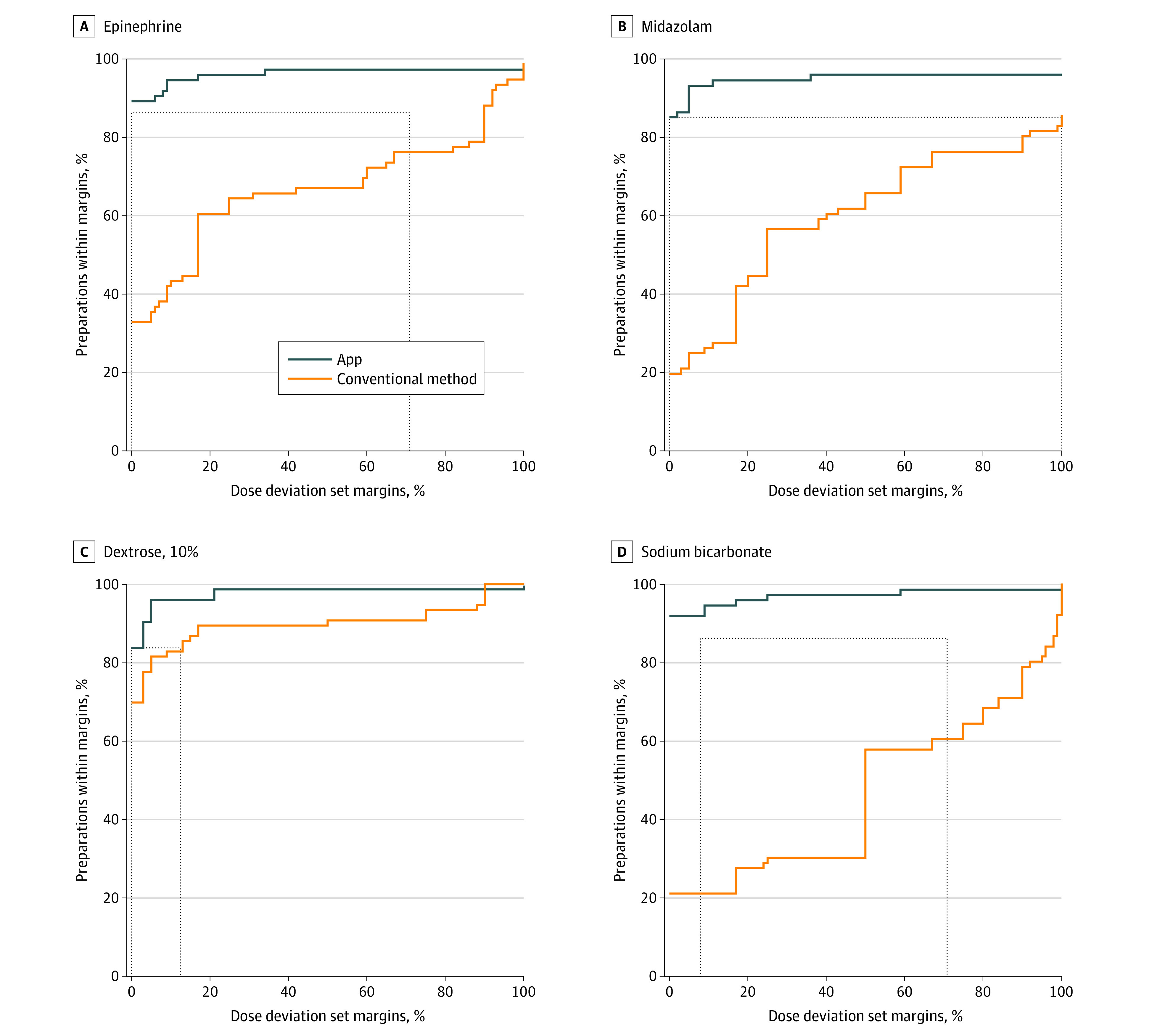
Proportions of Drug Doses Within Dose Deviation Set Margins From Prescribed Doses for Each of the 4 Drugs Curves represent the percentage of preparations (y-axis) with a dose deviation (underdose or overdose) lower than a specified margin when this margin ranged from 0% to 100% of the prescribed dose (x-axis). Dashed horizontal line indicates the percentage of preparations with a dose deviation of 0% in the app group; and vertical dashed lines, dose deviation set margin that should be accepted in the conventional method group to achieve this percentage. For example, in panel A, a dose deviation set margin of 91.7% would categorize 89.2% of epinephrine preparations via the conventional method as acceptable and 100% of epinephrine preparations via the app as acceptable. Thus, for this example, even with a tolerable limit of dose deviation set high at 91.7% of the prescribed dose, 10.8% of errors would still occur with the conventional method.

For the first drug (epinephrine), the dose deviation was 0% for 66 delivered doses (89.2%) with the app, but up to 92% for 70 delivered doses (92.1%) with the conventional method. For the frequency of drug deviations, a dose deviation set margin of 0% with the app was similar to a dose deviation set margin of 92% with the conventional method (Figure 2). Similar results were found for the second (midazolam) and fourth drugs (sodium bicarbonate), and the equivalent dose deviation set margin for the third drug (10% dextrose) was 13% (Figure 2).

### Secondary Outcomes

Significantly shorter times to drug preparation and delivery were observed for 3 of the drugs when using the app (eTable 5 in Supplement 2). Overall, with the app, time to drug preparation decreased by 40 seconds (95% CI, 23-57 seconds; *P* < .001), and time to drug delivery decreased by 47 seconds (95% CI, 27-66 seconds; *P* < .001). Compared with the conventional method, these decreases when using the app represented an overall savings in time of 20%, with the greatest time savings for the fourth and hardest-to-prepare drug (sodium bicarbonate) (34% decrease).

The annual number of critical pediatric cases handled by the EMS and paramedics’ years of practice did not modify the intervention effect (eTable 6 in Supplement 2). The variability of individual recorded preparation and delivery times was lower with the app than with the conventional method (eFigure 3 in Supplement 2).

## Discussion

In this multicenter, randomized clinical trial, medication error rates were significantly lower with the use of a custom-designed mobile app than with the use of conventional methods for the prehospital preparation of 4 drugs for direct intravenous administration by paramedics. To date, there is a paucity of studies providing insight into the magnitude of error related to drug preparations for direct intravenous administration during pediatric out-of-hospital medical situations in critical care, and errors are underreported in this setting.^2,5^ Reasons for a high likelihood of these errors may be the limited number of paramedics, the inherent complexity of preparing drugs at pediatric doses under stress, and the considerable time constraints. In this trial, drug dose deviations were the main cause of medication errors when conventional methods were used, with epinephrine administered beyond the proper dose range in proportions up to 60%. These results are consistent with those of previous studies.^9,31^

The risk of incorrect preparations varied across drugs when using the conventional methods, with a higher risk for drugs that were more difficult to prepare or less frequently used; however, the risk did not vary when using the app. The consistent decrease in risk to a low level of approximately 5% for all 4 drugs with use of the app regardless of their varying degrees of drug preparation difficulty may reflect the influence of the app on securing the preparation stage of the medication process irrespective of the context. This stage is particularly prone to medication errors when multiple steps are required, with each step being a potential source of error and especially when the task is cognitively loaded and uncommon.^6,7,32^ Pediatric situations account for approximately 7% of EMS calls, with delivery of epinephrine to children accounting for only 3.6% of the total adult drug administration.^33^ In many critical situations, paramedics are still dependent on conventional paper-based support, empirical calculators, height and weight estimation tapes (eg, Broselow-Luten or Handtevy tapes), or spreadsheets to ensure correct drug delivery. However, controversy remains over the accuracy of these tools to function as an effective resuscitation aid for the prevention of prehospital medication errors.^34,35^ In 2019, the US National Highway Traffic Safety Administration released their vision for the future of pediatric prehospital care to be achieved by 2050 and advocated for alternative approaches that do not require EMS personnel to calculate medication doses.^36^ One solution may be to use prefilled, weight-based, color-coded syringes, but these are not yet commercially manufactured with standardized pediatric volumes.^37,38^ Another innovative solution may be to use a syringe holder kit as a substitute (eg, Certa Dose^39^), although this is currently limited to only a few drugs and is not evidence based. To date, no app designed to assist in pediatric drug preparation at the point of care has been validated in the out-of-hospital setting.^12^ The present trial suggests that a mobile app such as PedAMINES may meet these expectations in a lightweight, affordable, and scalable manner to support emergency drug preparation at the point of care. The development of PedAMINES also contributes to the goals of the World Health Organization’s third Global Patient Safety Challenge, which has the aim to decrease severe, avoidable medication-associated harm by 50% in all countries during the next 5 years.^3^

Early administration of epinephrine was highlighted as one of the major updates to the 2020 American Heart Association guidelines.^40^ Most patients in the prehospital setting receive epinephrine more than 10 minutes after EMS arrival.^41^ Although the survival rate has numerous complex components, every minute saved in the preparation of emergency medications in the prehospital setting may lead to an increase in the odds of survival of 9%.^41^ In the present trial, the use of the app invariably decreased the mean time to each drug delivery. The magnitude of time reduction appeared to be inversely associated with drug preparation habits, suggesting a greater benefit of use of the app for infrequent preparations. This result was observed irrespective of paramedic years of experience or the annual number of critical pediatric cases handled by the EMS. The ability to decrease the delay to drug delivery from the moment the drug is prescribed may contribute to improved patient survival.

### Limitations

This study has limitations. First, the use of a simulated setting may be criticized. However, high-fidelity simulation is an essential method to assess research questions and technology that cannot be addressed during real-world cardiopulmonary resuscitation because, in addition to ethical issues, heterogeneity among patients and their diseases makes such studies difficult to standardize in critical situations. Second, the threshold of 10% drug dose deviation used to define a medication error may seem to be both conservative and potentially of limited clinical consequence. Although dose deviation ranges and their clinical influence in resuscitation studies are not evidence based, this threshold was recognized by the most recent expert consensus on principles and thresholds of pediatric dosing in critical care medicine.^25^ In the present trial, even when setting higher thresholds up to a 50% set margin, medication errors remained significantly higher without the app. Third, the 5-minute app training was dispensed immediately before the scenario. In real-life situations, the interval between training and actual use may be months. However, providing individuals with training for the app months before the study would have informed them of the purpose of the app and may have created a preparation bias. Fourth, only 4 drugs were used in this trial, but these drugs were a representative sample of the difficulty levels that may be encountered in the preparation of other emergency drugs. The results obtained with these 4 drugs suggest a benefit of the use of the app by paramedics to similarly decrease the rate of medication errors with other emergency drugs.

## Conclusions

In this randomized clinical trial, fewer medication errors and shorter times to drug delivery for the direct intravenous administration of emergency drugs in the prehospital setting were observed when paramedics used a mobile app designed to help pediatric drug preparation compared with conventional methods. Because potentially harmful medication errors are frequent, this trial suggests that dedicated medical mobile apps have the potential to improve medication safety and change prehospital clinical practice in pediatric emergency medicine. Because trial interpretation is limited by the simulation-based design, a next step may be to determine in real-life studies whether the decreased rate of medication errors and time saved owing to the use of this app translates into similar results in clinical practice.

## Notes

Supplement 1.
Trial Protocol

Supplement 2.
eMethods.
**eFigure 1.** Pediatric Accurate Medication in Emergency Situations (PedAMINES) App Screenshot **eFigure 2.** Bland and Altman Analysis of Video Review for Time to Drug Preparation and Time to Drug Delivery **eFigure 3.** Boxplots of Time to Drug Preparation and Time to Drug Delivery for Participants Using the Mobile App Compared With Use of the Conventional Preparation Method **eTable 1.** Interrater Agreement on Medication Errors Analysis **eTable 2.** Dose Deviation by Incremental Set Margins, per Drug **eTable 3.** Details of Drug Over- and Underdoses per Drug and Study Arm Expressed as a Median Percentage Deviation From the Prescribed Dose **eTable 4.** Details of Medication Errors With the App **eTable 5.** Differences in Outcomes **eTable 6.** Subgroup Analysis for Primary and Secondary Outcomes by Years Since Paramedic Certification and EMS Annual Number of Pediatric Interventions
eReferences.

Supplement 3.
The Pediatric Accurate Medication in Emergency Situations (PedAMINES) Prehospital Group

Supplement 4.
Data Sharing Statement
