# Supplementary material for: Effect of a Mobile App on Prehospital Medication Errors During Simulated Pediatric Resuscitation: A Randomized Clinical Trial
Source: JAMA Netw Open. 2021 Aug 30;4(8):e2123007. doi: 10.1001/jamanetworkopen.2021.23007 (PMC8406083; doi:10.1001/jamanetworkopen.2021.23007)
Supplement: Supplement 1. — Trial Protocol [file jamanetwopen-e2123007-s001.pdf]

Effect of a Mobile App on Prehospital Medication Errors During Simulated Pediatric Resuscitation: A Randomized Clinical Trial

Study Protocol

National Clinical Trial (NCT) Identifier Number: NCT03921346.
Registered on April 18, 2019.
Principal Investigator: Johan N. Siebert (Johan.Siebert@hcuge.ch)
Funded by: Swiss National Science Foundation
Fund Number: SNSF_32003B_182374
Version Number: v.1.0
June 10, 2021

<u>Important</u>

According to Art. 47 paragraphs 2–4 of the Swiss Federal Act on Research involving Human Beings (Human Research Act, status as of 1 January 2014), and Swiss Ethics Committees on research involving humans (Swissethics), multicenter studies that are being conducted in several cantons/trial centers in Switzerland should not have to be reviewed in full by every cantonal ethics committee, but only from the lead ethics committee which is responsible at the site of activity of the project coordinator. This original protocol for this trial received a declaration of no objection by the Geneva Cantonal Ethics Committee on 29 March 2018, as the purpose of the study was to examine the effect of the intervention on health care providers.

The final version of the protocol was published online November 20, 2019 at https://doi.org/10.1186/s13063-019-3726-4. This reformatted version of the study protocol was created on June 10, 2021 at the request of the reviewing journal.

ROLES and RESPONSIBILITIES:

Principal Investigator:

Signed: 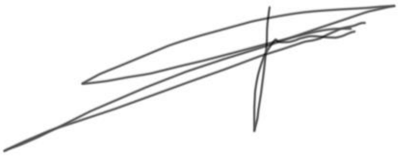 Date: June 10, 2021

Name: Johan N. Siebert
Title: MD, Deputy Head

Investigator contact information:

Affiliation: Children's Hospital, Geneva University Hospitals
Address: 47 Avenue de la Roseraie, 1205 Geneva, Switzerland
Phone: +41 79 55 34 072
Email: Johan.Siebert@hcuge.ch

Sponsor (within the meaning of the Swiss Ordinance on Clinical Trials in Human Research of 20 September 2013, Article 2):

None

1. Introduction

1.1 Background

Children are a vulnerable population with specific medical needs compared to adults. The fast, accurate and safe preparation and administration of intravenous (IV) drugs is both complex and time-consuming in pediatric critical situations, such as cardiopulmonary resuscitation (CPR)[1-4]. Most drugs given intravenously to children are provided in vials originally prepared for the adult population. This leads to the need for a specific individual, weight-based drug dose calculation and preparation for each child that varies widely across age groups[3, 5-9]. This error-prone process and the lower dosing error tolerance of children[10] place them at a high risk for life-threatening medication errors[3, 5, 6, 11]. Despite well-equipped and staffed environments with numerous available safeguards, direct IV medication errors have been reported in up to 41% of cases during simulated in-hospital pediatric resuscitations, 65% of which were incorrect medication dosage, thus making it the most common error[12]. The rate of errors is also important in the high-risk prehospital setting, which is reported as occurring in more than 30% of all pediatric drugs administered, with an error rate for epinephrine dosage alone of more than 60%[13]. In this particular context, initial care has to be delivered quickly by emergency medical services (EMS) in challenging field environments where resources and providers are limited[14]. A single paramedic is often in charge of determining the patient's weight, choosing the most suitable drug, calculating the drug dose and appropriate volume to inject, and administering it to the patient. However, as paramedics have little exposure to pediatric education during their initial training[15] and thereafter to critically ill children during their work hours[16], they have limited opportunities to administer resuscitation drugs at pediatric doses and to improve their skills level.

In resuscitation, time is a critical success criterion. During the first 15 min of pediatric CPR, survival and favorable neurological outcome decrease linearly by 2.1% and 1.2% per min, respectively[17], and rely in part on drug preparation time both in-[18] or out-of-hospital settings[19]. Among non-shockable pediatric out-of-hospital cardiac arrests, each minute delay to epinephrine delivery is associated with a 9% decrease in survival odds[19, 20]. Regrettably, most patients in the prehospital setting receive epinephrine more than 10 min after EMS arrival[19, 20]. Therefore, the chain of survival critically relies on early out-of-hospital CPR by EMS[21] and onsite administration of emergency drugs without delay[19, 20, 22] before a rapid transfer to pediatric emergency departments (PED) and advanced care. Despite efforts to solve this problematic, out-of-hospital preparation and delivery of pediatric emergency drugs remain a worldwide health challenge. The evaluation of new methods to reduce pediatric medication errors is of paramount importance, but research in this area is scarce.

1.2 Previous work justifying this trial

In a previous multicenter, randomized crossover trial, we showed that medication errors, time to drug preparation, and time to drug delivery for continuous infusions during simulation-based pediatric in-

hospital postcardiac arrest scenarios were significantly reduced by using a mobile device app (the pediatric accurate medication in emergency situations [PedAMINES]) designed to help pediatric drug preparation[23].

2. Methods

2.1 Objectives and hypothesis

The primary aim of this multicenter study protocol is to compare the impact of the app with conventional calculation methods for the preparation of direct IV drugs during standardized, simulation-based, pediatric out-of-hospital cardiac arrest scenarios.

We hypothesized that the use of the app might extend and scale-up our previous multicenter in-hospital observations by similarly reducing the occurrence of medication errors and time to drug preparation and delivery when used in out-of-hospital settings by paramedics, independent of EMS skills.

2.2 Trial design

We will conduct a prospective, multicenter, randomized controlled trial with two parallel groups in several EMS located in different regions of Switzerland, a pluralistic country with 4 official languages without uniformly standardized or benchmarked EMS clinical guidelines, protocols or operating procedures. Participants allocated to the conventional preparation method group will be allowed to use a calculator, but not any other drug preparation support enabling weight-based drug dose calculation, such as an online calculator or a mobile device app. The final correct volume of drugs to be drawn will not be released to the paramedics. To calculate the volume of drug to inject, the desired drug to be delivered in mg is first selected from a calculation of the original weight-based prescription in mg/kg. The next step is to convert the mg into mL of drug to be drawn. For the purpose of the study, we will not select drugs that can be directly drawn from the vial without calculation. Participants allocated to the app group will not be allowed to use any other drug preparation support.

Figure 1 shows the trial flow chart and **Figure 2** the trial checklist. The study will be carried out in accordance with the Consolidated Standards of Reporting Trials of Electronic and Mobile Health Applications and Online TeleHealth (CONSORT-EHEALTH)[24] guidelines and the Reporting Guidelines for Health Care Simulation Research [25]. The present study protocol adheres to the SPIRIT 2013 Checklist[26].

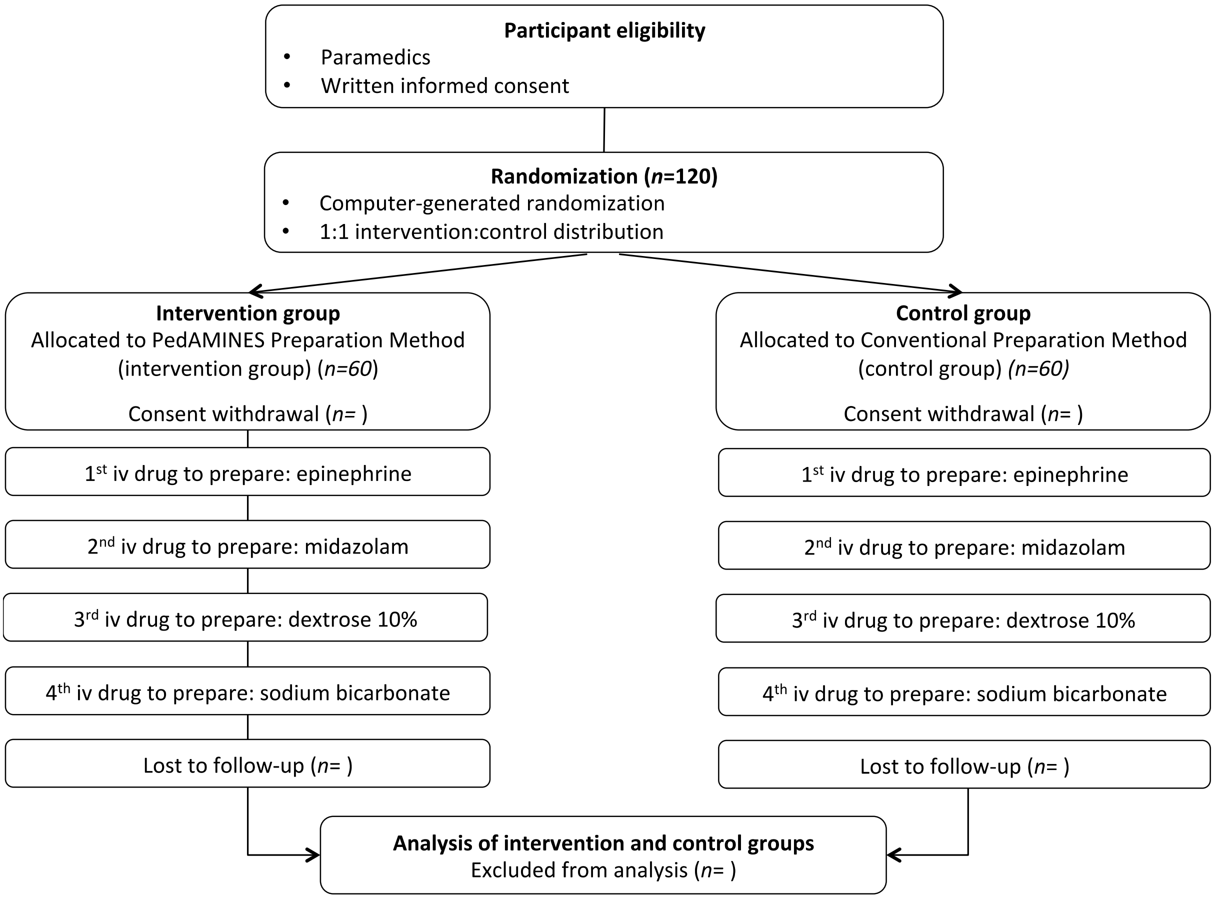

Figure 1. Trial flow chart.

The trial protocol received a declaration of no objection by the Geneva Cantonal Ethics Committee on March 29, 2018. The trial will be conducted according to the principles of the Declaration of Helsinki[27] and Good Clinical Practice guidelines[28]. Results will be reported according to recommendations in the CONSORT-EHEALTH Statement[24] and the Reporting Guidelines for Health Care Simulation Research[25]. It is our intention to present these at scientific congresses and to publish the results in an international peer-reviewed journal, irrespective of the magnitude or direction of effect.

2.3 Participants

Registered paramedics working in Swiss EMS are eligible for inclusion in the study. Inclusion criteria are having followed a standardized 5-min introductory course on the use of the mobile device app and willing to grant written informed consent. They will be excluded if they had previously used a numerical device aimed at helping with drug preparation. All participants will be assumed to have an equivalent competence with direct IV drug preparation and dose calculation as this is part of their regular practice and training background.

2.4 Choice of comparator: the PedAMINES app

The app was developed at Geneva University Hospitals (Geneva, Switzerland) following a user-centered and evidence-based approach with emergency department caregivers, software developers and ergonomists. On the basis of pediatric resuscitation observations and focus groups, the team worked closely together to identify the key functionalities and processes to be implemented[29]. The app lists all the available resuscitation drugs for either direct IV injection or continuous infusion with doses automatically adapted to the weight or age of the patient based on information entered when starting the app. With one touch, any of the listed drugs can be selected and shown with a detailed preparation according to a standardized and simplified pathway. In the case of a direct IV injection, this pathway is composed of two steps: 1) drug selection and 2) conversion of the prescribed dose in mg/kg into a volume in mL. If necessary, an additional step is provided for the dilution of the initial drug concentration with compatible fluids (sodium chloride 0.9%, etc.). For each drug, the exact amount to prepare is clearly displayed and thus avoids the need for calculations. This is based on the app’s ability to automatically calculate the optimal weight-based final volume to inject and describe the preparation sequence required to achieve it, independent of the user’s competency in this domain. When using the app, the user can interact with it at any time. Multiple drugs can be prepared and run in parallel, including continuous infusions. All actions by the user are sequentially saved locally on the device in historic files to preserve information that can be retrieved at any time for debriefing or medicolegal purposes. Historic files can also be erased or safely exported and saved in electronic health records.

2.5 Intervention and resuscitation scenario

On the day of participation after random allocation, each participating paramedic will: 1) complete a survey collecting data regarding their demographics, care training, and simulation and computer experience; 2) receive a standardized 5-min training session on how to use PedAMINES; as well as 3) a presentation of the simulation manikin characteristics. The paramedics will then be asked to perform a 20-min highly realistic pediatric CPR scenario on a high-fidelity WiFi manikin (Laerdal SimBaby, Laerdal Medical, Stavanger, Norway). The procedure will be standardized across all sites to follow the same chronological progression and range of difficulty in order to ensure that each participant is exposed to exactly the same case, with similar challenges in decision-making and treatment preparation provided on the same manikin. The uniform delivery of the scenario throughout the entire study will minimize confounders. Study team members will only adapt to the progression speed of participants through the scenario by maintaining a stressful resuscitation atmosphere. The scenario will be conducted in an out-of-hospital simulated child bedroom environment to increase realism. High levels of realism are known to immerse participants in the simulated experience and prevent confounding variables that might potentially affect the way individuals perform[30]. The room will be exclusively devoted to the simulation to prevent unexpected interruptions or external stimuli. Portable

monitoring alarms will be activated to increase realism and stress. The scenario will be filmed with 3 action video cameras (GoPro, Hero 5 Black edition; San Matteo, CA, USA) worn by the participating paramedic and placed within the room.

The untimed portion of the simulation will involve a resuscitation team comprised of the same 2 study team members throughout the whole study period. One member (LB) will play the role of a second paramedic leading the CPR and assisting the study participant by performing chest compressions and bag-valve mask ventilation, but not drug dose calculation or preparation, and the other (JS) will have the role of an advanced life support physician as part of the responding crew, but supposedly dispatched to the scene in a second phase to prescribe the emergency drugs. In several countries, physicians are an integral part of prehospital EMS teams and are often dispatched to the most severe cases, including cardiac arrest[31]. Drug prescription will comply with standard international pediatric life-saving doses. A certified technician (SM) will operate the simulator and play the role of the patient's father, supposedly devoid of resuscitation knowledge and competencies. Participants will be informed before the scenario starts that these 3 people are study team members. The second paramedic will guide each participant through a series of predefined key steps, blinded to the participant, following a standardized resuscitation scenario (see below). The physician will order sequentially the medications using International Nonproprietary Names and allow progression through the scenario only once predefined milestones have been reached, irrespective of error occurrence or the time taken to achieve them. Study-specific training and standardization of the second paramedic and physician is ensured through their involvement in the previous in-hospital studies[23, 32] and by following the predefined scenario.

The untimed portion of the simulation will start by turning on the 3 video cameras and a fitness watch on the participant's wrist, with both paramedics waiting outside the room. Both will be invited to enter the child's bedroom by the patient's father. When entering the room, a clinical statement to recognize the life-threatening condition of the patient, including his exact weight and age, will be given by the father as follows: "Here is Junior, a 12-kg, 18-month-old boy who suddenly collapsed 15 minutes ago. Oral pills belonging to his grandmother were found in his mouth and on the floor of his room. He is unconscious, pale and not breathing". Looking at the empty medicine boxes, the second paramedic says "that the pills are an oral tricyclic antidepressant, as well as antidiabetic medication". At this moment, the second paramedic says "OK, I'll take the lead of the resuscitation" and asks the participant to take a central pulse. Due to the invariable absence of a pulse, the participant is asked to assist the leader in doing a 2-min full-course massage and ventilation (30:1 ratio) maneuver, with the massage carried out by the participant to increase his/her stress level. During this time, the leader places a supraglottic airway device in the manikin's throat and the defibrillator patches on the trunk. The physician then enters the room and an asystole rhythm is recognized and verbalized. Both the

physician and leader rotate the person performing the massage-ventilation maneuvers (new 15:2 ratio), ask the participant to place a vascular access on the manikin's right hand (not intra-osseous to preserve the manikin integrity; first IV attempt successful) and then to prepare the drugs.

On the basis of the American Heart Association pediatric cardiac arrest algorithm for asystole[33], a bolus of 0.01 mg/kg epinephrine (0.1 mL/kg of 0.1 mg/mL concentration) is ordered by the physician and the timed scenario begins. The participating paramedic must prepare and administer the drug with the help of the app (intervention group) or following the conventional calculation method. The return of spontaneous circulation ensues. At this time, an upper-arm blood pressure monitor, a digital pulse oximeter on the right index finger, and a capnography on a bag-valve mask are placed on the manikin who suddenly begins to have generalized tonic-clonic seizures. The physician says "the patient has now a return of spontaneous circulation with a pulse, but with seizures" and tells the participant "this patient needs a direct IV bolus of 0.1 mg/kg midazolam (of 5 mg/mL concentration ad 10 mL sodium chloride 0.9%) right now", while the leader is invited to stop the massage-ventilation maneuvers. The seizures stop 15 sec after administration of the drug. At this time, the physician asks the leader to perform a fingerstick to obtain a blood sample. The glucometer reports a blood sugar of 0.8 mmol/L. The physician says "the patient has a severe hypoglycemia" and prompts the participant to prepare and inject a direct IV bolus of 4 mL/kg dextrose 10%. Return of a state of consciousness ensues with normal vital signs, but a wide QRS on electrocardiogram monitoring. The physician says "this child needs a direct IV bolus of 1 mmol/kg sodium bicarbonate (of 4.2% = 0.5 mmol/mL concentration)". As soon as this last medication is administered, the physician asks for transport to advanced hospital care and the scenario ends. The GoPro cameras and the watch are turned off 1 min later.

During the timed scenario, the resuscitation team will maintain a stressful resuscitation atmosphere by frequently reporting vital signs aloud and asking the participant to promptly provide the drugs, the monitoring alarms will be turned on, and the father will repeatedly verbalize his dismay. The measured deviation between the amount of drug delivered and the actual prescribed dose will be measured by the amount of drug in the syringe and video recorded. All usual EMS resuscitation equipment will be at the disposal of the paramedic. In both allocation groups, the decision to use or not use any equipment will remain personal as in real life. Neither pilot testing nor repetitions will be permitted. There will be no interventions or educational adjuncts prior to or after the study period. To ensure that participants hear and understand the prescription orders correctly and to avoid comprehension bias, they will have to confirm the orders verbally and written transcriptions will be checked and video-recorded. Immediately after the scenario, participants will be asked to recall and describe precisely how they had prepared the drugs and to complete a questionnaire about the scenario.

2.6 Outcomes

The primary outcome will be the proportion of medication dosage containing errors that occur during the sequence from drug preparation to drug injection. We define an emergency medication dose administration error as a deviation from the correct weight dose of more than 10%[7, 34]. These errors will be measured both as the percentage deviation from the amount of delivered drug compared with the correct weight dose as prescribed by the physician and the absolute deviations from that dose. Miscalculation of the final drug amount, the inability to calculate drug dosage without calculation and guidance help from the second paramedic, and deviation of more than 10% of the final administered concentration of sodium bicarbonate from the prescribed 4.2% will also be considered as medication errors.

The secondary outcome will be the elapsed time in seconds between the oral prescription by the physician and time to drug preparation completion by the participant, the elapsed time in seconds between the oral prescription by the physician and time to drug delivery by the participant (both times being relevant temporal values described in the pediatric resuscitation literature)[17, 19, 35], and analysis of the type of medication error (i.e., error in transcription of the physician's order into the medication dose, wrong choice of drug, wrong vial's initial concentration, wrong air purge out of the syringe before injection, stage of error detection [before or after injection], and aseptic errors). These measures of medication errors have been selected as they were considered to be the most commonly reported in the pediatric medication error literature and a meta-analysis[36-38]. In addition, a 3-item questionnaire using a 10-point Likert scale will be administered to participants. The questionnaire measures: 1) the stress perceived before the scenario starts ("on a scale from 1 to 10, how stressed are you now?"); 2) the overall stress perceived at the end of the scenario ("on a scale of 1 to 10, how stressed [maximum reached] were you during the drug preparation period?"); and 3) the satisfaction about the preparation method used during the resuscitation scenario ("on a scale of 1 to 10, how satisfied were you with your preparation experience?").

Participants' self-assessed psychological stress will be measured using Gauthier and Bouchard's French-Canadian adaptation[39] of Spielberger's psychometric State-Trait Anxiety Inventory test (STAI) questionnaire[40, 41]. Perceived stress will be also assessed by self-assessment using a numerical 10-point Likert visual analogue scale (VAS)[42]. The participants' physiologic stress level will be assessed by measuring their heart rate (HR) (see below).

Acceptability and usability testing of the app will be assessed using a 52-item questionnaire based on the unified theory of acceptance and use of technology (UTAUT) model[43]. The UTAUT is a

standardized instrument for measuring the likelihood of success of new technology introductions and helps to understand the drivers of its acceptance.

2.7 Methods of measurement and data collection

Research using simulation as a valid and reliable investigative methodology to study factors affecting human and systems performance in health care has been reviewed[30]. In this study, all actions (i.e., outcomes) performed by the paramedics during the scenario will be automatically recorded and stored by the responsive simulator detectors (Laerdal SimBaby) and the 3 GoPro video cameras. The set-up of the 3 cameras will be standardized to record at a resolution of 1080p at 90 frames per sec, a wide field of view, and a 16:9 aspect ratio. Similarly, the position of the cameras will be standardized. The first camera will be mounted on a head strap placed on the paramedic's head with a 45° downward inclination to allow to capture footage of the front scene. The second camera will be placed on a tripod in front of the paramedic and the manikin, slightly above head height, with a 90° downward inclination to film the place where the drugs will be prepared. The third camera will be placed on a tripod 1 m away from the paramedic on his/her left (if right-handed) or right (if left-handed) at the navel level to film the scene from the side. The recorded videos will be safely stored in triplicate on secured hard disk drives, kept in a locked room, and centralized at the Children's Hospital in Geneva. As all scenarios will be fully video-recorded, medication errors and any other errors will be recorded and later analyzed.

All actions performed with the app will be automatically saved locally in log files for further analysis. The validity and reliability of the app has been assessed in prior studies[23, 32]. STAI and VAS questionnaires will be used to measure perceived stress. HR will be measured as a surrogate of physiologic sympathetic response to stress[44]. A single continuous measurement at 1-second intervals will be recorded during the scenario with optical photoplethysmography using a Polar A360 wrist-worn HR monitor (Polar Electro Oy, Kempele, Finland). Data locally stored on the wristwatch itself during the scenarios will be synchronized with the dedicated Polar FlowSync web service for latter offline analyses. Several time-points of cardiovascular reactivity will be measured: 1) the minimal HR measured within the 5 min before the scenario starts ($HR_{baseline}$) while participants are not performing mental or physical exercise, 2) peak HR (HR_{peak}) for each drug, defined as the maximal HR reached during the sequence from drug prescription by the physician to drug delivery, 3) increased percentage of HR change for each drug by substracting $HR_{baseline}$ from corresponding HR_{peaks} [$((HR_{peak}-HR_{baseline})/HR_{baseline})*100$]. An additional $HR_{recovery}$ will also be measured as the minimal HR measured during the 5 minutes immediately following scenario completion (i.e., at the stop of the timed period of the scenario represented by patient's arousal). The investigators will double-check on-site that the questionnaires are fully and accurately completed. Data collection will be carried out using the REDCap database (REDCap, Vanderbilt University, Nashville, TN, USA). This study offers the major

advantage to observe a unique 60-min period per paramedic. Therefore, neither follow-up nor retention plans will be necessary. The intervention protocol is highly standardized and paramedic deviation from the protocol in terms of drug preparation is a parameter that is of interest in our study (i.e., in terms of medication errors or delays in drug preparation).

2.8 Power and sample size calculation

The expected proportion of errors made by EMS without PedAMINES is 60%[6]. The sample size was calculated to provide the trial with 90% power at a two-sided alpha level of 5% in detecting an absolute difference of at least 30% in proportions of medication errors between intervention groups. The required sample size is 56 paramedics per study arm. To prevent a potential loss of power due to misspecification of assumptions, 60 paramedics will be recruited per randomized group (total sample size: 120 paramedics). To achieve adequate participant enrolment to reach the target sample size, shift-working paramedics will be randomly recruited weeks before the start of the study by a blinded non-investigator. They will be informed of the upcoming simulation study but not of its purpose and outcomes.

2.9 Group allocation

Paramedics will be randomized using a stratified, single, constant 1:1 allocation ratio determined with web-based software[45]. One randomization list per EMS center will be produced (randomization stratified on center) and random block sizes will be used to generate the randomization lists. On the day of participation, each participant and an investigator will sign informed consent, and selection criteria will be checked prior to participation in the study.

2.10 Blinding

Blinding to the direct IV drugs and doses intended for use will be maintained during recruitment to minimize preparation bias. Allocation concealment will be ensured with the allocation software and will not be released until the paramedics start the scenario. Study team members will be revealed to the participants just before the scenario starts. Although the intervention could not be masked, all investigators will remain unaware of the outcomes until all data are unlocked for analysis at the end of the trial. All scenarios will be video-recorded for later analysis. Post-scenario video review will be done without blinding by two reviewers, but undertaken independently with each blinded to the other’s reviews. In the case of disagreement, a third independent evaluator will help reach a consensus.

2.11 Confidentiality

Information about study subjects will be kept confidential. All data will be entered into the REDCap data management system where all data on study subjects are assigned an individual identifying code that does not contain identifying information.

2.12 Statistical analysis plan

Planned statistical analyses will be published with the protocol. Due to the study population and interventions, we did not anticipate any missing data. The full analysis of the case will be the primary analysis. In the case of missing data, a sensitivity analysis will be performed by replacing the missing data with a multiple imputation procedure.

The overall risk of medication errors (primary outcome) related to the four drugs will be compared between study arms by using a logistic regression model with mixed effects. A random intercept will be introduced in the model with a random effect to account for the repetition of measures within paramedics. We plan to adjust for the center (fixed effect) since the randomization will be stratified on centers. Additionally, an adjustment for the type of medication is planned. In case of low number of paramedics or lack of medication errors when using the app in some centers, the center and the type of drug will be introduced as random effects instead of fixed effects. All random effects will be crossed. The intervention (app versus conventional methods) will be set as a fixed effect. With this model, the regression coefficients of fixed effects will model the risk of medication errors for a typical paramedic, a typical center, and a typical drug (that is when all random effects are set to 0). Therefore, the estimates of the odds ratio (OR) for the app's effect can be different from the OR calculated with raw data. As recommended by the CONSORT 2010 guidelines,[46] an additive measure of the effect size will be assessed. For this purpose, a parametric bootstrap approach will be used: 1) the risk of medication in both arms predicted by the model will obtained from the regression coefficients (fixed effects) by using the inverse of the logit transformation and the risk difference will be calculated; 2) 100,000 sets of regression coefficients will be randomly generated from a multivariable normal distribution with the estimates of the fixed effects as mean and the covariance matrix of the estimates as variance; 3) the risk difference will be obtained for each generated set by using the inverse of the logit transformation as in step 1; 4) the percentile 95% confidence interval (CI) will be obtained from the 100,000 risk differences calculated in step 3.

A similar approach is planned to assess the effect size of the app on the components of the medication error (dose deviation > 10%, help required). In case of an absence of assistance from the paramedic investigator, and therefore an absence of convergence of the model, the risk difference will be assessed using Miettinen-Nurminen's approach[47] to account for the stratified randomization. With this approach, the repetition of measures within paramedics (outcome assessed on four drugs) will not be accounted. The third component (drug concentration deviation > 10%) will also be investigated by using a logistic regression model with mixed effects. Since this component will be assessed only for the fourth drug (sodium bicarbonate), a single random effect (center) will be introduced in the model. A similar approach will be used to assess the app's effect on medication errors (primary outcome) per

drug. Only the P-value for testing the app's effect on the medication errors over the four drugs obtained with the logistic regression model will be reported. The analyses of the components of the primary outcome and the analyses per drugs aim to better characterise the app's effect. The 95% CIs of the effect sizes will not be adjusted for multiple analyses.

Secondary outcomes, the time to drug preparation and the time to drug delivery, will be analyzed using linear regression models with mixed effects. We plan to adjust for center and the type of drugs (fixed effects). Pre-specified sub-group analyses will be performed by introducing an interaction term in the regression models with mixed effects.

Dose deviations will be investigated: for each drug, the frequency of under- and overdoses will be assessed as well as the median (interquartile interval) relative dose deviations. The cumulative distribution of the absolute value of the relative dose deviation will be graphically represented. The distribution of the relative dose deviation will be compared between study arms by using Van Elteren's test stratified on centers.

For primary and secondary outcomes, logistic regression analyses will be conducted if applicable to test a difference in error rates between an urban EMS (defined as a primary EMS in an area populated with 50,000 or more people in the immediate proximity of a tertiary care PED)[48] and a rural EMS (EMS agency not included within an urban area) with the app and conventional methods. In a generalized estimating equation (GEE) logistic regression model, an interaction between interventions and urban/rural EMS will be tested to investigate a potential modification of the efficacy of the app in an urban area compared with a rural area. Results will be also correlated to the EMS exposure (i.e., total number of emergency calls per year per EMS divided by the number of paramedics working in that EMS). Analyses of primary and secondary outcomes will be also conducted with both preparation methods according to paramedics' experience, expressed as years since certification.

A first reviewer will review all videos. To assess the reproducibility of the video review procedure, a second reviewer will independently duplicate the review in a random sample of 10% of all videos. Interrater reliability scores on video reviewing will be calculated using Cohen's kappa coefficient for the medication errors. As the other outcomes are continuous variables, the Bland-Altman method will be used to plot the difference of values reported by both reviewers against the mean value for each outcome. The limits of agreement will be assessed by the interval of ±1.96 standard deviations (SD) of the measurement difference either side of the mean difference. The null hypothesis that there is no difference on average between both reviewers will be tested using a t-test. The mean difference will be reported with its 95% CI. Additionally, the intraclass correlation coefficients for volumes of drugs drawn, time to drug preparation, and time to drug delivery will be assessed, assuming that raters are a

sample from a larger population of possible raters. The agreement will be investigated for the data of each study period.

Finally, means and SDs will be determined for perceived stress and satisfaction scores of individuals for each questionnaire item, as well as for the UTAUT questionnaire, and reported with descriptive statistics. Pearson correlations will be computed between the HR measures obtained with the watch and the scenario phases for each of the drugs and preparation methods used. In the case of missing data, a complete case analysis will be conducted. No multiple imputations are planned. All statistical tests will be two-sided with a type one error risk of 5%. All statistical analyses will be performed with R software (version 4.0; Foundation for Statistical Computing), and with GraphPad Prism (version 7.0; GraphPad Software) for graph figures.

2.13 Criteria for discontinuing or modifying interventions

The time needed to complete the full scenario was set to only a 20-minute period for each participant and will be booked several weeks in advance to ensure availability of the participants without repercussions on their normal duty. All participants will receive full permission from their hierarchy to participate to the study. Using a simulation-based CPR scenario makes our study highly feasible without any risks to patients or participants.

2.14 Adherence

The study will take place during a one-week period in each EMS and will occupy each paramedic for a single 60-minute period. Due to our experience gathered from previous studies, we anticipate a very low rate of drop-outs or loss of follow-up. To ensure the presence of participants on the day of participation, shift-working paramedics will be randomly recruited 1 month before the start of the study by a blinded non-investigator. They will be informed of the upcoming simulation study, but not of its purpose and outcomes. Adherence of the collaborating EMS to our study will be reinforced by their citation in further publications and a privileged access to the PedAMINES app.

2.15 Participants timeline

On-site staff in each collaborating EMS will organize the participation of paramedics to the study. No information about the ongoing study will be given to paramedics at this stage. The best study period will be matched with study coordinators. The paramedics will be spread across one week in order to link up the several scenarios. On scheduled weeks, the study coordinators will visit the collaborating EMS, provide trial information to participants before they begin the scenario, and assessment of eligibility will be verified. Participants will then sign the informed consent form. They will be asked to complete an anonymized demographic questionnaire and confirm their willingness to participate. If this is the case, they will pursue with a 5-minute educational session given by the investigators on the

use of PedAMINES. Each participant will then be randomly allocated to the study arms. Immediately after randomization, the paramedic will start the scenario. The total study period to enrol all paramedics in the various EMS will take 12 months.

	STUDY PERIOD				
	Pre-enrolment	Enrolment	Pre-study baseline / allocation	20-min scenario	Close-out
**TIMEPOINT**	$-t_2$	$-t_1$	t_0	t_1	t_x
**STUDY PROCEDURES**					
Invitation to participate	✓				
Recruitment		✓			
Eligibility screening		✓			
Informed consent form		✓			
PedAMINES 5-min learning session		✓			
Randomization			✓		
**INTERVENTION**					
Intervention group (with PedAMINES)				✓	
Control group (with conventional methods)				✓	
**ASSESSMENTS**					
Case report form (demographic + clinical data)			✓		
STAI and VAS questionnaires			✓		
Medication errors				✓	
Time at drug prescription				✓	
Time to drug preparation				✓	
Time to drug delivery				✓	
Continuous HR recording with Polar A360				✓	
STAI and VAS questionnaires					✓
3-item satisfaction termination questionnaire					✓
Adapted UTAUT questionnaire					✓

Figure 2. Standard Protocol Items: Recommendations for Interventional Trials checklist (SPIRIT) figure.

2.16 Recruitment

We already have the study participation approval of the different EMS. Each collaborating EMS will organize a dedicated time slot for the study to be run and for paramedics to be available at the defined period. In the case of the unavailability of a paramedic at the selected date, the collaborating EMS will handle the procedure to replace the paramedic. The investigators and collaborating EMS will be in close contact by telephone/email to guarantee the availability of the paramedics. The number of available paramedics will be guaranteed by the collaborating EMS given their knowledge of their total workforce and the paramedics' work shifts. Given the calculated sample size, the number of paramedics participating from each EMS can be managed without any detrimental impact on service provision.

2.17 Interim analysis

We will not perform interim analyses as our trial has a short duration and no potential serious outcomes.

2.18 Study schedule and milestones

<table>
<tr><td>Preparation phase</td><td colspan="2">Study year 1</td><td>Study year 2</td></tr>
<tr><td rowspan="2">Milestones

Study preparation
Duration: 2 months
- Workshops with EMS heads to prepare the study venue in their centers
- On-site collaborating EMS visits to prepare the study venue (material and drug availability, paramedic availability, etc.)
- Invitation to paramedics to participate to the study
- Wireless manikin SimBaby™ and accessories purchased and available
- Data management system ready
- Web-based centralized randomization ready (www.sealedenvelope.com)
- Transport for amenities and investigators booked
- Hotels for investigators booked</td><td colspan="2">Milestones

Study conduct
Duration: 12 months

Including:
- All participant enrollment and participation: 1 week per EMS center
- Data management: 2 weeks per collaborating EMS
- 20% extra time for unexpected incidents (technical incident, paramedic unavailability, etc.)</td><td rowspan="2">Milestones

Data analysis
Duration: 4 months

- Video reviewing and analysis
- Data management

Statistics
Duration: 1 month full time

Study closure
Workshop with study partners

Write-up of the study
Duration: 5 months
- Final analysis of endpoints
- Writing of manuscripts

Dissemination
Publications, congresses, and networking</td></tr>
<tr><td>Number of participants included after 12 months:</td><td>120 (full enrollment completed)</td></tr>
</table>

2.19 Date of completion of preparation phase

01.01.2019

References

1. Brindley PG, O'Dochartaigh D, Volney C, Ryan S, Douma MJ. Time delays associated with vasoactive medication preparation and delivery in simulated patients at risk of cardiac arrest. *J Crit Care*. 2017;40:149-153. doi:10.1016/j.jcrc.2017.04.003

2. Flannery AH, Parli SE. Medication errors in cardiopulmonary arrest and code-related situations. *Am J Crit Care*. 2016;25(1):12-20. doi:10.4037/ajcc2016190

3. Kaufmann J, Laschat M, Wappler F. Medication errors in pediatric emergencies: a systematic analysis. *Dtsch Arztebl Int*. 2012;109(38):609-616. doi:10.3238/arztebl.2012.0609

4. Moreira ME, Hernandez C, Stevens AD, et al. Color-coded prefilled medication syringes decrease time to delivery and dosing error in simulated emergency department pediatric resuscitations. *Ann Emerg Med*. 2015;66:97-106. doi:10.1016/j.annemergmed.2014.12.035

5. Gonzales K. Medication administration errors and the pediatric population: a systematic search of the literature. *J Pediatr Nurs*. 2010;25(6):555-565. doi:10.1016/j.pedn.2010.04.002

6. Hoyle JD, Davis AT, Putman KK, Trytko JA, Fales WD. Medication dosing errors in pediatric patients treated by emergency medical services. *Prehosp Emerg Care*. 2012;16(1):59-66. doi:10.3109/10903127.2011.614043

7. Marcin JP, Dharmar M, Cho M, et al. Medication errors among acutely ill and injured children treated in rural emergency departments. *Ann Emerg Med*. 2007;50(4):361-367, 367 e1-2. doi:10.1016/j.annemergmed.2007.01.020

8. Morgan N, Luo X, Fortner C, Frush K. Opportunities for performance improvement in relation to medication administration during pediatric stabilization. *Qual Saf Health Care*. 2006;15(3):179-183. doi:10.1136/qshc.2005.017350

9. Stucky ER, American Academy of Pediatrics Committee on Drugs, American Academy of Pediatrics Committee on Hospital Care. Prevention of medication errors in the pediatric inpatient setting. *Pediatrics*. 2003;112(2):431-6.

10. Kaushal R, Bates DW, Landrigan C, et al. Medication errors and adverse drug events in pediatric inpatients. *JAMA*. 2001;285(16):2114-1120. doi:10.1001/jama.285.16.2114

11. Kamarainen A. Out-of-hospital cardiac arrests in children. *J Emerg Trauma Shock*. 2010;3(3):273-276. doi:10.4103/0974-2700.66531

12. Porter E, Barcega B, Kim TY. Analysis of medication errors in simulated pediatric resuscitation by residents. *West J Emerg Med*. 2014;15(4):486-490. doi:10.5811/westjem.2014.2.17922

13. Hoyle JD, Jr., Crowe RP, Bentley MA, Beltran G, Fales W. Pediatric prehospital medication dosing errors: a national survey of paramedics. *Prehosp Emerg Care*. 2017;21(2):185-191. doi:10.1080/10903127.2016.1227001

14. Cottrell EK, O'Brien K, Curry M, et al. Understanding safety in prehospital emergency medical services for children. *Prehosp Emerg Care*. 2014;18(3):350-358. doi:10.3109/10903127.2013.869640

15. Su E, Schmidt TA, Mann NC, Zechnich AD. A randomized controlled trial to assess decay in acquired knowledge among paramedics completing a pediatric resuscitation course. *Acad Emerg Med*. 2000;7(7):779-786. doi:10.1111/j.1553-2712.2000.tb02270.x

16. Shah MN, Cushman JT, Davis CO, Bazarian JJ, Auinger P, Friedman B. The epidemiology of emergency medical services use by children: an analysis of the National Hospital Ambulatory Medical Care Survey. *Prehosp Emerg Care*. 2008;12(3):269-276. doi:10.1080/10903120802100167

17. Matos RI, Watson RS, Nadkarni VM, et al. Duration of cardiopulmonary resuscitation and illness category impact survival and neurologic outcomes for in-hospital pediatric cardiac arrests. *Circulation*. 2013;127(4):442-451. doi:10.1161/CIRCULATIONAHA.112.125625

18. Andersen LW, Berg KM, Saindon BZ, et al. Time to epinephrine and survival after pediatric in-hospital cardiac arrest. *JAMA*. 2015;314(8):802-810. doi:10.1001/jama.2015.9678

19. Hansen M, Schmicker RH, Newgard CD, et al. Time to epinephrine administration and survival from non-shockable out-of-hospital cardiac arrest among children and adults. *Circulation*. 2018;137(19):2032-2040. doi:10.1161/CIRCULATIONAHA.117.033067

20. Fukuda T, Kondo Y, Hayashida K, Sekiguchi H, Kukita I. Time to epinephrine and survival after paediatric out-of-hospital cardiac arrest. *Eur Heart J Cardiovasc Pharmacother*. 2018;4(3):144-151. doi:10.1093/ehjcvp/pvx023

21. Foltin GL, Richmond N, Treiber M, et al. Pediatric prehospital evaluation of NYC cardiac arrest survival (PHENYCS). *Pediatr Emerg Care*. 2012;28(9):864-868. doi:10.1097/PEC.0b013e3182675e70

22. Rittenberger JC, Bost JE, Menegazzi JJ. Time to give the first medication during resuscitation in out-of-hospital cardiac arrest. *Resuscitation*. 2006;70(2):201-206. doi:10.1016/j.resuscitation.2005.12.006

23. Siebert JN, Ehrler F, Combescure C, et al. A mobile device application to reduce medication errors and time to drug delivery during simulated paediatric cardiopulmonary resuscitation: a multicentre, randomised, controlled, crossover trial. *Lancet Child Adolesc Health*. 2019;3(5):303-311. doi:10.1016/S2352-4642(19)30003-3

24. Eysenbach GC-EG. CONSORT-EHEALTH: improving and standardizing evaluation reports of web-based and mobile health interventions. *J Med Internet Res*. 2011;13(4):e126. doi:10.2196/jmir.1923

25. Cheng A, Kessler D, Mackinnon R, et al. Reporting guidelines for health care simulation research: extensions to the CONSORT and STROBE statements. *Simul Healthc*. 2016;11(4):238-248. doi:10.1097/SIH.0000000000000150

26. Chan AW, Tetzlaff JM, Gotzsche PC, et al. SPIRIT 2013 explanation and elaboration: guidance for protocols of clinical trials. *BMJ*. 2013;346:e7586. doi:10.1136/bmj.e7586

27. World Medical Association. Declaration of Helsinki: ethical principles for medical research involving human subjects. JAMA 2013; 310(20): 2191-2194.

28. International Conference on Harmonisation. Harmonised tripartite guideline. Statistical principles for clinical trials. International conference on harmonisation E9 expert working group. *Stat Med*. 1999;18(15):1905-1942.

29. Hagberg H, Siebert J, Gervaix A, et al. Improving drugs administration safety in pediatric resuscitation using mobile technology. *Stud Health Technol Inform*. 2016;225:656-657.

30. Cheng A, Auerbach M, Hunt EA, et al. Designing and conducting simulation-based research. *Pediatrics*. 2014;133(6):1091-1101. doi:10.1542/peds.2013-3267

31. Bottiger BW, Bernhard M, Knapp J, Nagele P. Influence of EMS-physician presence on survival after out-of-hospital cardiopulmonary resuscitation: systematic review and meta-analysis. *Crit Care*. 2016;20:4. doi:10.1186/s13054-015-1156-6

32. Siebert JN, Ehrler F, Combescure C, et al. A mobile device app to reduce time to drug delivery and medication errors during simulated pediatric cardiopulmonary resuscitation: a randomized controlled trial. *J Med Internet Res*. 2017;19(2):e31. doi:10.2196/jmir.7005

33. de Caen AR, Berg MD, Chameides L, et al. Part 12: Pediatric advanced life support: 2015 American Heart Association guidelines update for cardiopulmonary resuscitation and emergency cardiovascular care. *Circulation*. 2015;132(18 Suppl 2):S526-S542. doi:10.1161/CIR.0000000000000266

34. Roumeliotis N, Pullenayegum E, Rochon P, Taddio A, Parshuram C. A modified Delphi to define drug dosing errors in pediatric critical care. *BMC Pediatr*. 2020;20(1):488. doi:10.1186/s12887-020-02384-3

35. Banerjee PR, Ganti L, Pepe PE, Singh A, Roka A, Vittone RA. Early on-scene management of pediatric out-of-hospital cardiac arrest can result in improved likelihood for neurologically-intact survival. *Resuscitation*. 2019;135:162-167. doi:10.1016/j.resuscitation.2018.11.002

36. Kozer E, Seto W, Verjee Z, et al. Prospective observational study on the incidence of medication errors during simulated resuscitation in a paediatric emergency department. *BMJ*. 2004;329(7478):1321. doi:10.1136/bmj.38244.607083.55

37. Maaskant JM, Vermeulen H, Apampa B, et al. Interventions for reducing medication errors in children in hospital. *Cochrane Database Syst Rev*. 2015;3:CD006208. doi:10.1002/14651858.CD006208.pub3

38. Rinke ML, Bundy DG, Velasquez CA, et al. Interventions to reduce pediatric medication errors: a systematic review. *Pediatrics*. 2014;134(2):338-360. doi:10.1542/peds.2013-3531

39. Gauthier J, Bouchard S. A French-Canadian adaptation of the revised version of Spielberger's State–Trait Anxiety Inventory. *Can J Behav Sci*. 1993;25(4):559-578. doi:10.1037/h0078881

40. State-Trait Anxiety Inventory Web site. Accessed June 8, 2021. http://www.mindgarden.com/products/staisad.htm

41. Spielberger CD. Manual for the State-Trait Anxiety Inventory STAI (form Y) (" self-evaluation questionnaire"). 1983. Accessed June 8, 2021. https://ubir.buffalo.edu/xmlui/handle/10477/1873

42. Lesage F-X, Berjot S, Deschamps F. Clinical stress assessment using a visual analogue scale. *Occup Med*. 2012;62(8):600-605. doi:10.1093/occmed/kqs140

43. Venkatesh V, Morris MG, Davis GB, Davis FD. User acceptance of information technology: toward a unified view. *MIS Quarterly*. 2003;27(3):425-478. doi:10.2307/30036540

44. Furedy JJ, Szabo A, Peronnet F. Effects of psychological and physiological challenges on heart rate, T-wave amplitude, and pulse-transit time. *Int J Psychophysiol*. 1996;22(3):173-183. doi:10.1016/0167-8760(96)00025-6

45. http://www.sealedenvelope.com. Last accessed: 2018.10.30. Archived by WebCite. http://www.webcitation.org/6pj1kvMba

46. Schulz KF, Altman DG, Moher D, Group C. CONSORT 2010 statement: updated guidelines for reporting parallel group randomised trials. *BMJ*. 2010;340:c332. doi:10.1136/bmj.c332

47. Miettinen O, Nurminen M. Comparative analysis of two rates. *Stat Med*. 1985;4(2):213-226. doi:10.1002/sim.4780040211

48. Bureau USC. URL: https://www.census.gov/programs-surveys/geography/guidance/geo-areas/urban-rural/2010-urban-rural.html. Accessed: 2018-11-16.
