# Supplementary material for: Effect of a Mobile App on Prehospital Medication Errors During Simulated Pediatric Resuscitation: A Randomized Clinical Trial
Source: JAMA Netw Open. 2021 Aug 30;4(8):e2123007. doi: 10.1001/jamanetworkopen.2021.23007 (PMC8406083; doi:10.1001/jamanetworkopen.2021.23007)
Supplement: Supplement 2. — eMethods. eFigure 1. Pediatric Accurate Medication in Emergency Situations (PedAMINES) App Screenshot eFigure 2. Bland and Altman Analysis of Video Review for Time to Drug Preparation and Time to Drug Delivery eFigure 3. Boxplots of Time to Drug Preparation and Time to Drug Delivery for Participants Using the Mobile App Compared With Use of the Conventional Preparation Method eTable 1. Interrater Agreement on Medication Errors Analysis eTable 2. Dose Deviation by Incremental Set Margins, per Drug eTable 3. Details of Drug Over- and Underdoses per Drug and Study Arm Expressed as a Median Percentage Deviation From the Prescribed Dose eTable 4. Details of Medication Errors With the App eTable 5. Differences in Outcomes eTable 6. Subgroup Analysis for Primary and Secondary Outcomes by Years Since Paramedic Certification and EMS Annual Number of Pediatric Interventions eReferences. [file jamanetwopen-e2123007-s002.pdf]

Supplementary Online Content

Siebert JN, Bloudeau L, Combescure C, et al; Pediatric Accurate Medication in Emergency Situations (PedAMINES) Prehospital Group. Effect of a mobile app on prehospital medication errors during simulated pediatric resuscitation: a randomized clinical trial. *JAMA Netw Open*. 2021;4(8):e2123007. doi:10.1001/jamanetworkopen.2021.23007

eMethods.
eFigure 1. Pediatric Accurate Medication in Emergency Situations (PedAMINES) App Screenshot
eFigure 2. Bland and Altman Analysis of Video Review for Time to Drug Preparation and Time to Drug Delivery
eFigure 3. Boxplots of Time to Drug Preparation and Time to Drug Delivery for Participants Using the Mobile App Compared With Use of the Conventional Preparation Method
eTable 1. Interrater Agreement on Medication Errors Analysis
eTable 2. Dose Deviation by Incremental Set Margins, per Drug
eTable 3. Details of Drug Over- and Underdoses per Drug and Study Arm Expressed as a Median Percentage Deviation From the Prescribed Dose
eTable 4. Details of Medication Errors With the App
eTable 5. Differences in Outcomes
eTable 6. Subgroup Analysis for Primary and Secondary Outcomes by Years Since Paramedic Certification and EMS Annual Number of Pediatric Interventions
eReferences.

This supplementary material has been provided by the authors to give readers additional information about their work.

© 2021 Siebert JN et al. *JAMA Network Open*.

eMethods.

Resuscitation scenario

The scenario was conducted in an out-of-hospital, simulated, normal child's bedroom environment to increase realism. High levels of realism are known to immerse participants in the simulated experience and prevent confounding variables that might potentially affect the way individuals perform.[1] The room was exclusively devoted to the simulation to prevent unexpected interruptions or external stimuli. Portable monitoring alarms were activated to increase realism and stress.

The untimed portion of the simulation involved a resuscitation team comprised of the same two study team members throughout the whole study period. One member (LB) played the role of a second paramedic leading the cardiopulmonary resuscitation and assisting the study participant by performing chest compressions and bag-valve mask ventilation, but not drug dose calculation or preparation. The second member (JNS) had the role of an advanced life support physician as part of the responding crew, but supposedly dispatched to the scene in a second phase to prescribe the emergency drugs. In several countries, physicians are an integral part of prehospital emergency medical service (EMS) teams and are often dispatched to the most severe cases, including cardiac arrest.[2] Similar to the Anglo-American model,[3, 4] advanced paramedics in Switzerland constitute the initial response team and are qualified to independently prescribe a range of medications. In this study, resuscitation was led by a physician (JNS) in order to standardize the choice of drugs prescribed across the EMS and to avoid any deviation from the study protocol.

A certified technician (SM) operated the simulator and played the role of the patient's father, supposedly devoid of resuscitation knowledge and competencies. Participants were informed before the scenario start that these three people were study team members. The investigator paramedic guided each participant through a series of predefined key steps, blinded to the participant, following a standardized resuscitation scenario (see below). The physician ordered sequentially the medications using International Nonproprietary Names and allowed progression through the scenario only once predefined milestones had been reached, irrespective of error occurrence or the time taken to achieve them. Study-specific training and standardization of the second paramedic and physician was ensured through their involvement in the previous in-hospital studies[5, 6] and by following the predefined scenario. No conventional drug preparation training was provided in either group as this was part of the paramedics' daily practice. The intervention was standardized across all sites to follow the same chronological progression and range of difficulty in order to ensure that each participant was exposed to exactly the same case, with similar challenges in decision-making and treatment preparation provided on the same manikin. The uniform delivery of the scenario throughout the entire study was intended to minimize confounders. Study team members had only to adapt to the progression speed of participants through the scenario by maintaining a stressful resuscitation atmosphere.

The untimed portion of the simulation started by turning on the three video cameras and a Polar A360 wrist-worn heart rate monitor (Polar Electro Oy, Kempele, Finland), with both paramedics waiting outside the room. Both were invited to enter the child's bedroom by the patient's father. When entering the room, a clinical statement to recognize the life-threatening condition of the patient, including his exact weight and age, was given by the father as follows: "Here is Junior, a 12-kg, 18-month-old boy who suddenly collapsed 15 minutes ago. Oral pills belonging to his grandmother were found in his mouth and on the floor of his room". At this moment, the investigator paramedic using the pediatric assessment triangle to establish the child's clinical status says "OK, this child is unconscious, pale and not breathing. I'll take the lead of the resuscitation" and asks the participant to take a central pulse. Due to the invariable absence of a pulse, the participant is asked to assist the leader in doing a two-minute full-course massage and ventilation (30:1 ratio) maneuver, with the massage carried out by the participant to increase his/her stress level. During this time, the leader places a supraglottic airway device in the manikin's throat and the defibrillator patches on the trunk. Looking at the empty medicine boxes, the leader says that "the pills are an oral tricyclic antidepressant, as well as anti-diabetic medication". The physician then enters the room and an asystole rhythm is recognized and verbalized. Both the physician and leader rotate the person performing the massage-ventilation maneuvers (new 15:2 ratio), ask the participant to place a vascular access on the manikin's right hand (not intra-

© 2021 Seibert JN et al. *JAMA Network Open*.

osseous to preserve the manikin integrity; first intravenous (IV) attempt successful) and then to prepare the drugs.

On the basis of the American Heart Association pediatric cardiac arrest algorithm for asystole,[7] a bolus of 0.01 mg/kg epinephrine (0.1 mL/kg of 0.1 mg/mL concentration) is ordered by the physician and the timed scenario begins. The participating paramedic must prepare and administer the drug with the help of the app (intervention group) or following the conventional calculation method. The return of spontaneous circulation ensues. At this time, an upper-arm blood pressure monitor, a digital pulse oximeter on the right index finger, and a capnography on a bag-valve mask are placed on the manikin who suddenly begins to have generalized tonic-clonic seizures. The physician says “the patient has now a return of spontaneous circulation with a pulse, but with seizures” and tells the participant “this patient needs a direct IV bolus of 0.1 mg/kg midazolam (of 5 mg/mL concentration ad 10 mL sodium chloride 0.9%) right now”, while the leader is invited to stop the massage-ventilation maneuvers. The seizures stop 15 sec after administration of the drug. At this time, the physician asks the leader to perform a fingerstick to obtain a blood sample. The glucometer reports a blood sugar of 0.8 mmol/L. The physician says “the patient has a severe hypoglycemia” and prompts the participant to prepare and inject a direct IV bolus of 4 mL/kg 10% dextrose. Return of a state of consciousness ensues with normal vital signs, but with wide QRS signs of tricyclic antidepressant overdose on electrocardiogram monitoring. The physician says “this child needs a direct IV bolus of 1 mmol/kg sodium bicarbonate (of 4.2% = 0.5 mmol/mL concentration)”. As soon as this last medication is administered, the physician asks for transport to advanced hospital care and the scenario ends. The GoPro cameras and Polar A360 monitor are turned off 1 min later.

During the timed scenario, the resuscitation team maintained a stressful resuscitation atmosphere by frequently reporting vital signs aloud and asking the participant to promptly provide the drugs, the monitoring alarms were turned on, and the father repeatedly verbalized his dismay. All usual EMS resuscitation equipment was at the disposal of the paramedic. Participants allocated to the mobile app group (intervention group) were not allowed to use any other drug preparation support. Participants allocated to conventional methods (control group) were allowed to use a calculator, but not any other drug preparation support enabling weight-based drug dose calculation, such as an online calculator or a mobile device app. The app was at the disposal of the paramedics in their usual equipment and drug bag, already downloaded and ready to use, knowing that it natively boots natively in this operating mode. In both allocation groups, the decision to use or not use any equipment remained personal, as in real life. Neither pilot testing nor repetitions were permitted. There were no interventions or educational adjuncts prior to or after the study period. Immediately after the scenario, participants were asked to recall and describe precisely how they had prepared the drugs and to complete a 10-point Likert scale questionnaire about their perceived stress and satisfaction regarding the scenario.

Rationale for the selection of drugs

For the purpose of the study, no drugs that could have been directly drawn from the vial without calculation were selected. To calculate the volume of drug to inject, the desired drug to be delivered in mg was first selected from a calculation of the original weight-based prescription in mg/kg. The next step was to convert the mg into mL of drug to be drawn. Four IV drugs were chosen according to their use among paramedics and the difficulty of their preparation: epinephrine (1 mg/mL concentration), midazolam (5 mg/mL concentration), 10% dextrose (100 mg of dextrose/ml), and 8.4% sodium bicarbonate (84 mg of sodium bicarbonate/mL). These four drugs were a representative sample of the difficulty levels that may be encountered in the preparation of other emergency drugs. Drugs and diluent formulations used were those available to paramedics in their actual EMS systems. The chronology of their prescription along the scenario was established to follow a logical and credible clinical sequence of drug preparation as if they had to be prepared and administered in a real-life resuscitation situation. Thus, no presupposed difficulties in preparing these drugs were taken into consideration for their allocation order during the scenario. Drug prescriptions complied with standard international pediatric life-saving doses.

Epinephrine: epinephrine was chosen as it is the first drug of choice for both shockable and non-shockable cardiac arrests in adults and children and thus one of the most common drugs prepared by paramedics.[8] Its preparation requires a conversion from mg to ml and a supposedly simple one-step dilution prior to administration. For example, in this study a first standard-dose of epinephrine at 0.01

© 2021 Seibert JN et al. *JAMA Network Open*.

mg/kg IV was prescribed for a 12-kg patient (final dose of 0.12 mg). Epinephrine came in a 1.0 mg/mL initial concentration. Therefore, participants had first to draw up 1 mL in a 10-mL syringe and then to add 9 mL of 0.9% sodium chloride to the same 10-mL syringe (final concentration 0.1 mg/mL). They then had to deliver 1.2 mL intravenously.

Midazolam: midazolam was chosen because its use for prehospital seizure and acute agitation, although evidence-based, is reportedly underused by paramedics and therefore they are less familiar with its preparation than epinephrine.[9-11] For example, in a 12-kg patient, midazolam must be delivered at a final dose of 0.1 mg/kg (1.2 mg). Midazolam came in a 5 mg/mL initial concentration. Therefore, participants had to draw up 1 mL of midazolam and dilute it with 4 mL of 0.9% sodium chloride in a 5 mL syringe (1.0 mg/mL final concentration). They then had to deliver 1.2 mL intravenously.

Dextrose: 10% dextrose was supposedly the easiest drug to prepare as it is frequently used by paramedics and did not require conversion from mg to ml, but only a weight-based calculation in ml before to be drawn and injected. In a 12-kg patient, 10% dextrose must be delivered at a final dose of 4 mL/kg (48 mg). Dextrose came in a 50 g/500 mL initial concentration. Therefore, participants had to draw up 48 mL in a 50 mL syringe and deliver this volume intravenously (100 mg/mL final concentration).

Sodium bicarbonate: 4.2% sodium bicarbonate was anticipated to be the most complicated drug to prepare among the four. It was deliberately chosen as an "excuse drug" to evaluate the handling of a drug that is very rarely used in the prehospital setting (but which may prove necessary like other less commonly used drugs). Thus, its preparation to treat the patient’s tricyclic poisoning required: 1) the handling of a drug almost never used in the prehospital setting (but which could however prove necessary); 2) a weight-based calculation in mg before 3) a conversion into ml; and 4) the need to dilute the initial 8.4% hypertonic solution by half to 4.2% prior to injection to reduce the risk of extravasation-related tissue injury.[12, 13] In this study, for a 12-kg patient, 8.4% sodium bicarbonate (1000 mmol/L initial concentration; 84 mg/mL) was prescribed at 1 mmol/kg (12 mmol). Participants had first to draw up 12mL in a 50 mL syringe and dilute this solution by half to 4.2% (42 mg/mL) with 12 mL of 0.9% sodium chloride in the same 50 mL syringe. The then had to deliver the 24 mL intravenously.

The randomized design of this trial should prevent the occurrence of the under- or overestimation of errors in either group due to an inexperience in pediatric drug preparation for any of these four drugs.

Methods of measurement and data collection

Research using simulation as a valid and reliable investigative methodology to study factors affecting human and systems performance in health care has been reviewed.[1] In this study, all actions (that is, outcomes) performed by the paramedics during the scenario were automatically recorded and stored by the responsive simulator detectors (Laerdal New SimBaby, Laerdal Medical, Stavanger, Norway) and by three GoPro Hero 5 and 7 Black edition (GoPro Inc, San Matteo, CA, USA) video cameras. The set-up of the three cameras was standardized to record at a resolution of 1080p at 90 frames per second, a wide field of view, and a 16:9 aspect ratio. Similarly, the position of the cameras was standardized. The first camera was mounted on a head strap placed on the paramedic’s head with a 45° downward inclination to allow to capture footage of the front scene. The second camera was placed on a tripod in front of the paramedic and the manikin, slightly above head height, with a 90° downward inclination to film the place where the drugs were prepared. The third camera was placed on a tripod 1 m away from the paramedic on their left (if right-handed) or right (if left-handed) at navel level to film the scene from the side. The recorded videos were safely stored in triplicate on secured hard disk drives and kept in a locked cabinet in a secure location at the Children’s Hospital in Geneva. As all scenarios were fully video-recorded, medication errors and any other errors were recorded and later analyzed. To ensure that participants heard and understood the prescription orders correctly and to avoid comprehension bias, they had to confirm the orders verbally and written transcriptions were checked and video-recorded. The measured deviation between the amount of drug delivered and the actual prescribed dose was measured by the amount of drug in the syringe (i.e., volumes of drug and diluent), both through direct on-site visualization by one of the investigators (SM) during the scenario and a retrospective review of the video recordings after the scenario.

© 2021 Seibert JN et al. *JAMA Network Open*.

All actions performed with the app were automatically saved locally in log files for further analysis. The validity and reliability of the app has been assessed in prior studies.[5,6] The investigators double-checked on-site that the questionnaires were fully and accurately completed. Data collection was carried out using the REDCap database (REDCap, Vanderbilt University, Nashville, TN, USA). This study offered the major advantage to observe a unique 60-min period per paramedic. Therefore, neither follow-up nor retention plans were necessary. The intervention protocol was highly standardized and paramedic deviation from the protocol in terms of drug preparation was a parameter that was of interest in this study (i.e., in terms of medication errors or delays in drug preparation).

Other secondary outcomes were measured in this trial and will be analyzed and discussed in separate articles. These outcomes were the participants' stress level assessed subjectively by 1) pre-/post-intervention State Trait Anxiety Inventory questionnaires, and 2) self-reported perceived stress scores on a visual 10-point Likert analogue scale, as well as objectively by a single continuous measurement at 1-sec intervals as a marker of physiologic stress response, with optical heart rate measurements based on photoplethysmography using a Polar A360 wrist-worn heart rate monitor (Polar Electro Oy, Kempele, Finland). Another outcome will be the acceptability and usability testing of the app assessed using a technology acceptance, 26-item, self-administered survey slightly adapted from prior research[14] to fit the trial's context.

Ethics approval and consent to participate

According to Art. 47, paragraphs 2-4, of the Swiss Federal Act on Research involving Human Beings (Human Research Act, status as of 1 January, 2014), and Swiss ethics committees on research involving humans (SwissEthics), multicenter studies conducted in several cantons/trial centers in Switzerland should not have to be reviewed in full by every cantonal ethics committee, but only by the lead ethics committee responsible at the site of activity of the project coordinator. This trial received a declaration of no objection by the Geneva Cantonal Ethics Committee (Req-2019-00773) on 29 March, 2018, as the purpose of the study was to examine the effect of the intervention on healthcare providers. The trial was conducted according to the principles of the Declaration of Helsinki[22] and Good Clinical Practice guidelines.[23]

© 2021 Seibert JN et al. *JAMA Network Open*.

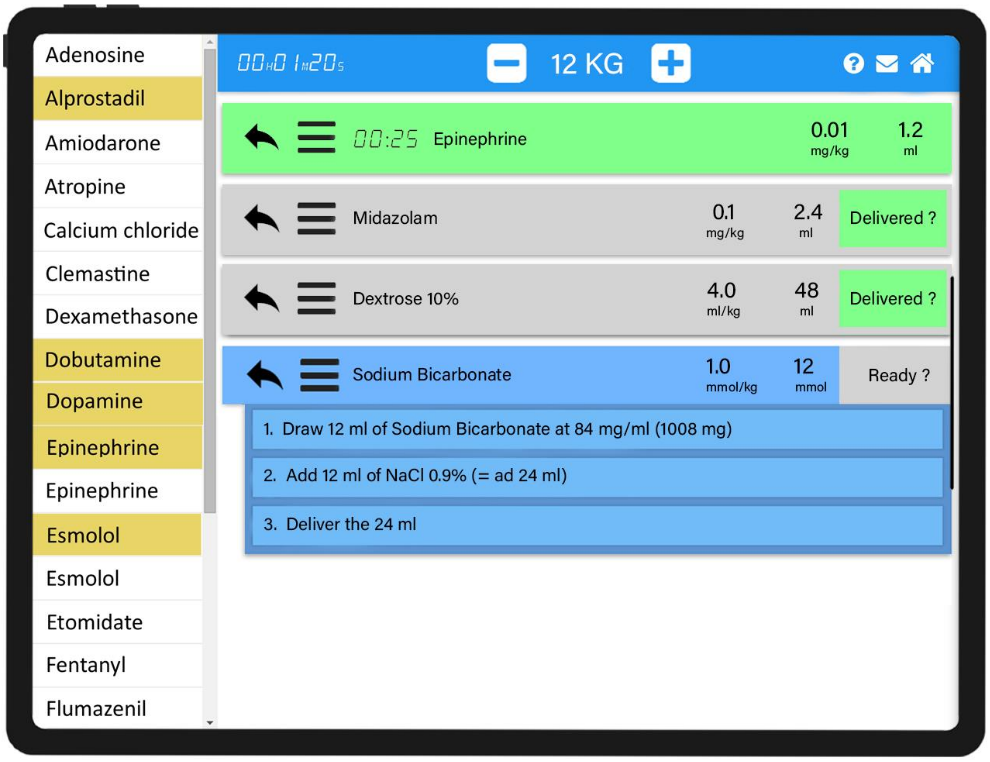

eFigure 1. Pediatric Accurate Medication in Emergency Situations (PedAMINES) App Screenshot

List of bolus IV-administered drugs (white boxes) and drugs for continuous infusion (yellow boxes) are selectable in the left margin of the app (only part of the list is shown in this figure due to the scrolling required to display the entire list). The right window shows drugs selected by the paramedic for a child weighing 12 kg. In this screenshot example, a bolus epinephrine is being delivered at 0.01 mg/kg (0.1 mL/kg of 0.1 mg/mL concentration). Midazolam 0.1 mg/kg (of 5 mg/mL concentration and 10 mL sodium chloride 0.9%) and dextrose 10% 4 mL/kg are selected and ready to be injected, waiting for the paramedic's approval ("delivered?"). Instructions to prepare sodium bicarbonate are displayed in a stepwise manner. The envelope logo in the upper right corner indicates that all actions performed by the paramedics are sequentially saved in historic files that can be retrieved, e-mailed, and printed at any time. The validity and reliability of the app has been assessed in prior studies.[5, 6]

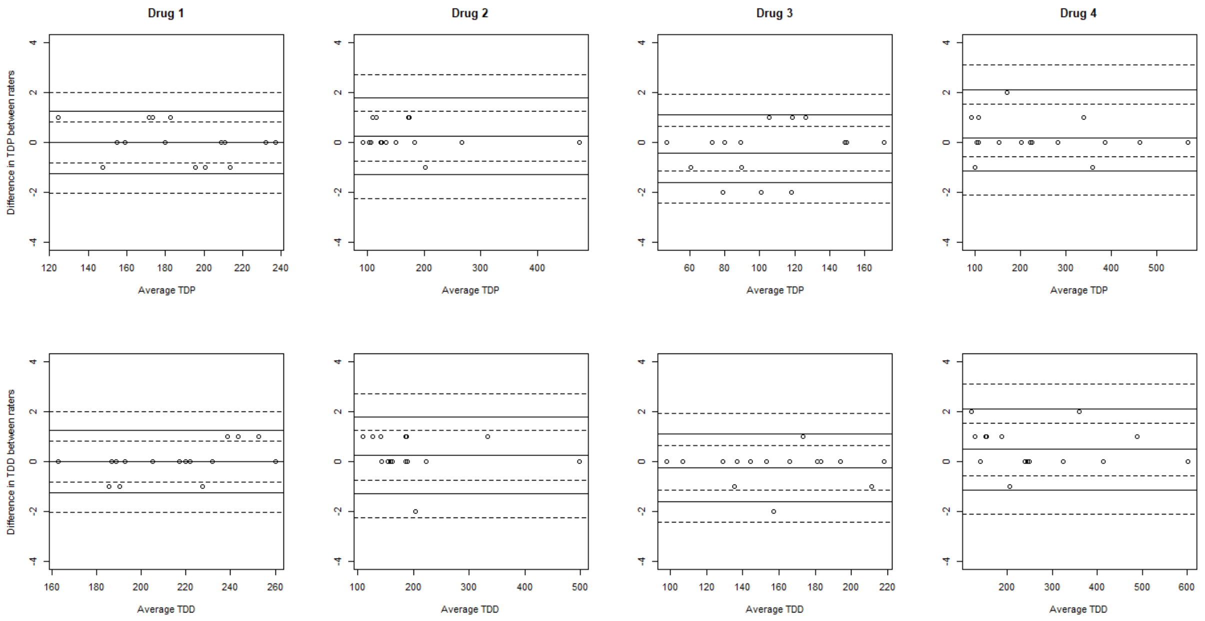

Outcome	Difference rater 1 - rater 2		Limits of agreement (95% CI)		ICC
	Mean (SD)	Min - Max	Lower	Upper	
**TDP**					
1st drug	0.0 (0.7)	-1 to 1	-1.5 (-2.3 to -0.9)	1.5 (0.9 to 2.3)	1
2nd drug	0.2 (0.6)	-1 to 1	-0.9 (-1.6 to -0.5)	1.4 (1.0 to 2.1)	1
3rd drug	-0.4 (1.1)	-2 to 1	-2.6 (-3.9 to -1.9)	1.7 (1.0 to 3.1)	1
4th drug	0.2 (0.8)	-1 to 2	-1.3 (-2.2 to -0.8)	1.7 (1.2 to 2.6)	1
**TDD**					
1st drug	0.0 (0.6)	-1 to 1	-1.3 (-2.0 to -0.8)	1.3 (0.8 to 2.0)	1
2nd drug	0.2 (0.8)	-2 to 1	-1.6 (-2.2 to -0.8)	1.8 (1.3 to 2.7)	1
3rd drug	-0.2 (0.7)	-2 to 1	-1.6 (-2.4 to -1.1)	1.1 (0.6 to 1.9)	1
4th drug	0.5 (0.8)	-1 to 2	-1.1 (-2.1 to -0.6)	2.1 (1.6 to 3.1)	1

eFigure 2. Bland and Altman Analysis of Video Review for Time to Drug Preparation and Time to Drug Delivery

The Bland-Altman plots illustrate the difference between reviewers 1 and 2 in TDP and TDD, plotted against the mean value of both reviewers. Solid lines denote the mean difference and the upper and lower limits of agreement (mean +/-1.96 SD of the difference). The dashed lines denote the 95% CIs of the limits of agreement.

1st drug: epinephrine; 2nd drug: midazolam; 3rd drug: 10% dextrose; 4th drug: sodium bicarbonate.
Abbreviations: TDP: time to drug preparation; TDD: time to drug delivery; SD: standard deviation; ICC: intraclass correlation coefficient; 95% CI: 95% confidence interval.

© 2021 Seibert JN et al. *JAMA Network Open*.

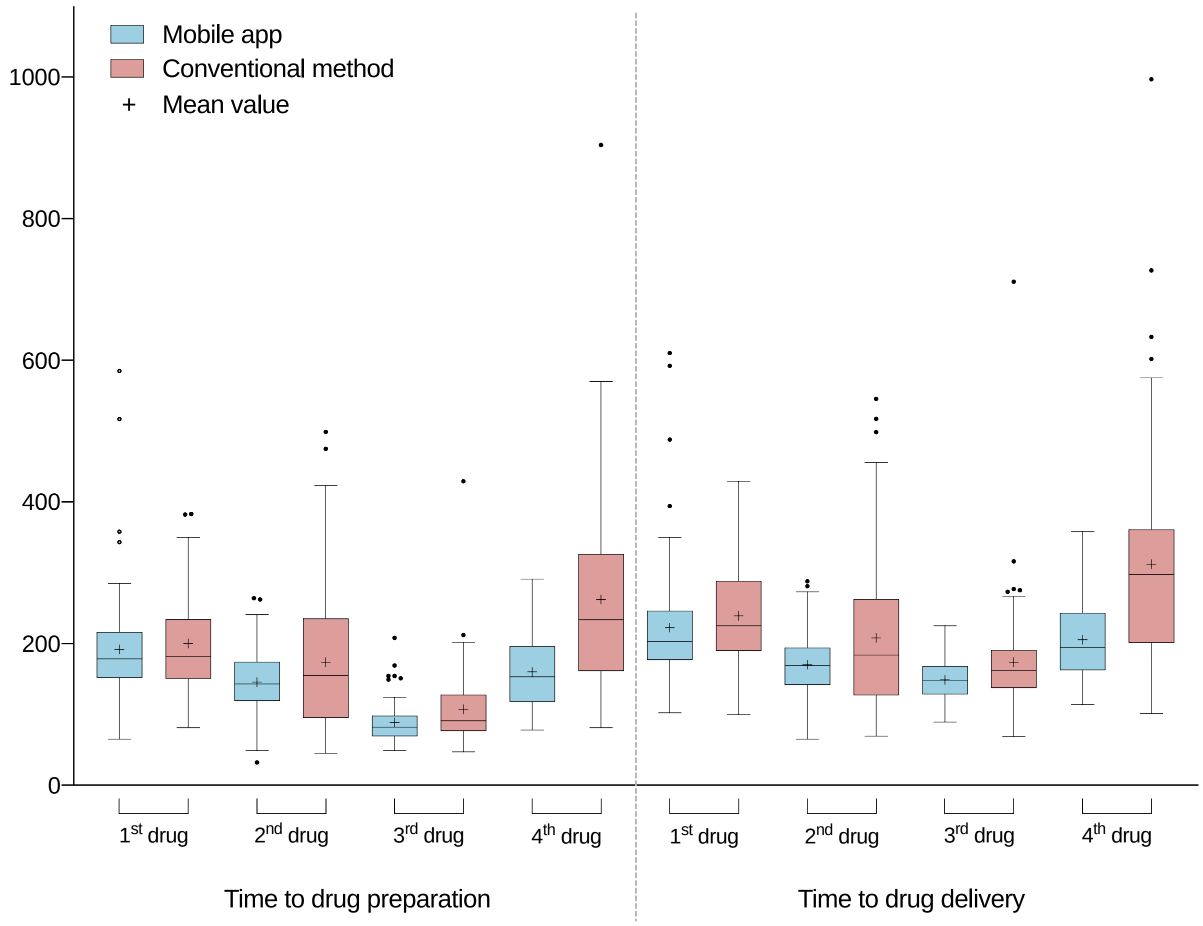

eFigure 3. Boxplots of Time to Drug Preparation and Time to Drug Delivery for Participants Using the Mobile App Compared With Use of the Conventional Preparation Method

Solid horizontal lines denote median and interquartile range; the endpoints of the whiskers indicate the range. The plus sign denotes the mean. Circles denote values that were more than 1.5 times the values represented at each end of the box. The long upper whiskers show that participants were more varied among the most positive quartile groups.
1st drug: epinephrine; 2nd drug: midazolam; 3rd drug: 10% dextrose; 4th drug: sodium bicarbonate.

© 2021 Seibert JN et al. *JAMA Network Open*.

eTable 1. Interrater Agreement on Medication Errors Analysis

Outcomes	Reviewer 1	Reviewer 2	Kappa coefficient
	n/N (%)	n/N (%)	(95% CI)
**1st drug**			
Requires help to prepare the	0/16 (0)	0/16 (0)	1 (NA)
Drug preparation error	4/16 (25.0)	4/16 (25.0)	1 (NA)
Total errors	4/16 (25.0)	4/16 (25.0)	1 (NA)
**2nd drug**			
Requires help to prepare the	2/16 (12.5)	2/16 (12.5)	1 (NA)
Drug preparation error	7/16 (43.8)	7/16 (43.8)	1 (NA)
Total errors	7/16 (43.8)	7/16 (43.8)	1 (NA)
**3rd drug**			
Requires help to prepare the	1/16 (6.3)	1/16 (6.3)	1 (NA)
Drug preparation error	1/16 (6.3)	1/16 (6.3)	1 (NA)
Total errors	2/16 (12.5)	2/16 (12.5)	1 (NA)
**4th drug**			
Requires help to prepare the	7/16 (43.8)	7/16 (43.8)	1 (NA)
Drug preparation error	8/16	7/16 (43.8)	0.875 (0.640-1.110)
Concentration error	6/16 (37.5)	6/16 (37.5)	1 (NA)
Total errors	9/16 (56.3)	9/16 (56.3)	1 (NA)
**Total**	10/64		1 (NA)
Requires help to prepare the	10/64 (15.6)	10/64 (15.6)	1 (NA)
Drug preparation error	20/64 (31.3)	19/64 (29.7)	1 (NA)
Total errors	22/64 (34.4)	22/64 (34.4)	1 (NA)

The table details the interrater agreement on video reviews by error type and drug, expressed as Cohen kappa coefficients. Poor reliability was defined as a kappa coefficient of <0.4, fair reliability as 0.4 to 0.6, good reliability as >0.6 to 0.8, and excellent as >0.8.
1st drug: epinephrine; 2nd drug: midazolam; 3rd drug: 10% dextrose; 4th drug: sodium bicarbonate.
Abbreviations: n: number of errors; N: number of preparation opportunities; 95% CI: 95% confidence interval

© 2021 Seibert JN et al. *JAMA Network Open*.

eTable 2. Dose Deviation by Incremental Set Margins, per Drug

	Dose deviation, n (%)				
**Set margin**	**Mobile app (N=74)**	**Conventional method (N=76)**	**Effect size (95% CI)**		
	n (%)	n (%)	Odds ratio	Risk difference	P-value
1st drug (epinephrine)					
10%	4 (5.4)	43 (56.6)	24.7 (7.8 to 78.0)	51.9 (37.2 to [illegible]	<.001
20%	3 (4.1)	30 (39.5)	15.7 (4.4 to 55.3)	35.4 (22.5 to [illegible]	<.001
30%	3 (4.1)	27 (35.5)	14.1 (3.9 to 50.3)	31.1 (18.2 to [illegible]	<.001
40%	2 (2.7)	26 (34.2)	20.7 (4.6 to 93.3)	30.7 (17.9 to [illegible]	<.001
50%	2 (2.7)	25 (32.9)	19.4 (4.3 to 87.5)	29.4 (16.8 to [illegible]	.001
2nd drug (midazolam)					
10%	5 (6.8)	56 (73.7)	38.6 (13.6 to [illegible]	66.9 (53.2 to [illegible]	<.001
20%	4 (5.4)	42 (55.3)	22.0 (7.1 to 68.3)	49.9 (35.9 to [illegible]	<.001
30%	4 (5.4)	33 (43.4)	14.7 (4.7 to 45.9)	38.1 (24.2 to [illegible]	<.001
40%	3 (4.1)	30 (39.5)	17.3 (4.8 to 62.3)	34.6 (21.1 to [illegible]	<.001
50%	3 (4.1)	26 (34.2)	12.3 (3.5 to 42.9)	30.2 (17.9 to [illegible]	<.001
3rd drug (10% dextrose)					
10%	3 (4.1)	13 (17.1)	5.0 (1.4 to 18.6)	12.2 (2.7 to [illegible]	.02
20%	3 (4.1)	8 (10.5)	2.8 (0.7 to 11.0)	6.4 (-2.8 to 17.8)	.14
30%	1 (1.4)	8 (10.5)	8.6 (1.0 to 45.9)	9.2 (0.3 to 18.1)	<.05
40%	1 (1.4)	8 (10.5)	8.6 (1.0 to 70.4)	9.2 (0.3 to 18.1)	<.05
50%	1 (1.4)	7 (9.2)	7.4 (0.9 to 61.7)	7.9 (-0.8 to 16.5)	.06
4th drug (sodium bicarbonate)					
10%	4 (5.4)	60 (78.9)	62.0 (17.6 to [illegible]	73.5 (60.4 to [illegible]	<.001
20%	3 (4.1)	55 (72.4)	62.0 (17.6 to [illegible]	68.3 (55.0 to [illegible]	<.001
30%	2 (2.7)	53 (69.7)	83.0 (18.7 to [illegible]	67.0 (53.7 to [illegible]	<.001
40%	2 (2.7)	53 (69.7)	83.0 (18.7 to [illegible]	67.0 (53.7 to [illegible]	<.001
50%	2 (2.7)	32 (42.1)	26.5 (6.0 to [illegible]	39.2 (26.1 to [illegible]	<.001

Abbreviations: n: number of errors; N: number of preparation opportunities; 95% CI: 95% confidence interval.

© 2021 Seibert JN et al. *JAMA Network Open*.

eTable 3. Details of Drug Over- and Underdoses per Drug and Study Arm Expressed as a Median Percentage Deviation From the Prescribed Dose

	Mobile App			Conventional Method		
	N	Median % deviation (IQR)	Min-max	N	Median % deviation (IQR)	Min-max
**1st drug**						
Underdoses	2	25.0 [20.8-29.2]	16.7-33.3	36	65.4 [16.7-90.0]	12.5-100.0
Overdoses	2	566.7 [400.0-733.3]	233.3-900.0	7	58.3 [33.3-63.3]	25.0-1150.0
**2nd drug**						
Underdoses	0	/	/	28	43.8 [16.7-58.3]	10.8-99.0
Overdoses	5	150.0 [35.8-200.0]	10.8-900.0	28	66.7 [25.0-308.3]	16.7-483.3
**3rd drug**						
Underdoses	3	20.8 [20.8-60.4]	20.8-100.0	10	81.3 [56.3-89.8]	12.5-90.0
Overdoses	0	/	/	3	14.6 [13.5-15.6]	12.5-16.7
**4th drug**						
Underdoses	3	25.0 [20.8-41.7]	16.7-58.3	56	62.5 [50.0-92.5]	16.7-99.9
Overdoses	1	100.0 [100.0-100.0]	100.0-100.0	4	70.8 [66.7-81.3]	66.7-100.0
**All drugs**						
Underdoses	8	22.9 [19.8-39.6]	16.7-100.0	130	58.3 [25.0-90.0]	10.8-100.0
Overdoses	8	175.0 [84.0-400]	10.8-900.0	42	63.3 [25.0-139.6]	12.5-1150

Abbreviations: IQR: interquartile range.
1st drug: epinephrine; 2nd drug: midazolam; 3rd drug: 10% dextrose; 4th drug: sodium bicarbonate.

© 2021 Seibert JN et al. *JAMA Network Open*.

eTable 4. Details of Medication Errors With the App

Participants	Details	Final volume and concentration administered
**1st drug: epinephrine 0.01 mg/kg (0.1 mL/kg of 0.1 mg/mL concentration)**		
Paramedic 1	Incorrect drug selection: click on epinephrine for continuous infusion. Realises the error before injection. Subsequently decides to proceed without the app. Rounds up the volume to inject	1 mL of 0.1 mg/mL epinephrine
Paramedic 2	Incorrect drug selection: click on epinephrine for continuous infusion	1 mL of 0.08 mg/mL epinephrine
Paramedic 3	App's instructions not followed	1.2 mL of pure 1 mg/mL epinephrine.
Paramedic 4	App's instructions followed for adenosine preparation, using epinephrine	0.4 mL of pure 1 mg/mL epinephrine
**2nd drug: midazolam 0.1 mg/kg (of 5 mg/mL concentration)**		
Paramedic 4	No dilution	2.4 mL of pure 5 mg/mL midazolam
Paramedic 5	Imprecise drug preparation (1.5 mL midazolam + 9.5 mL sodium chloride)	2.4 mL of 0.68 mg/mL midazolam
Paramedic 6	Dilutes the whole vial (3 mL) with 7 mL sodium chloride	2.4 mL of 1.5 mg/mL midazolam
Paramedic 7	Dilutes the whole vial (3 mL) with 7 mL sodium chloride	2.0 mL of 1.5 mg/mL midazolam
Paramedic 8	Inappropriate dilution (1 mL midazolam + 8 mL sodium chloride)	2.4 mL of 0.56 mg/mL midazolam
**3rd drug: dextrose 10% 4 mL/kg**		
Paramedic 2	Prepares and injects sodium bicarbonate instead of 10% dextrose, despite correct instructions on the app	48 mL of sodium bicarbonate 8.4%
Paramedic 9	Error in reading the graduation on the 50 mL syringe (38 mL instead of 48 mL)	38 mL of dextrose 100 mg/mL
Paramedic 10	Error in reading the app (38 mL instead of 48 mL)	38 mL of dextrose 100 mg/mL
**4th drug: sodium bicarbonate 1 mmol/kg (of 0.5 mmol/mL concentration = 4.2%)**		
Paramedic 1	Inappropriate dilution (5 mL sodium bicarbonate + 19 mL sodium chloride), despite correct instructions on the app	24 mL of 0.21 mol/L NaBic
Paramedic 11	Inappropriate dilution (9 mL sodium bicarbonate + 16 mL sodium chloride), despite correct instructions on the app	25 mL of 0.36 mmol/mL NaBic
Paramedic 12	No dilution	24 mL of pure NaBic
Paramedic 13	Inappropriate dilution when attempting to deliver the correct prescribed volume of drug into a small capacity syringe (i.e., 12 mL sodium bicarbonate + 8 mL sodium chloride in a 20 mL syringe)	20 mL of 0.6 mmol/mL NaBic
Paramedic 14	Imprecise drug preparation after air purge from the syringe (10 ml sodium bicarbonate + 14 mL sodium chloride)	24 mL of 0.42 mmol/mL NaBic
Abbreviation: NaBic: sodium bicarbonate.		

Details of the error committed by the paramedics for each drug. Paramedics may have committed errors in several medications. 1st drug: epinephrine; 2nd drug: midazolam; 3rd drug: 10% dextrose; 4th drug: sodium bicarbonate.

© 2021 Seibert JN et al. *JAMA Network Open*.

eTable 5. Differences in Outcomes

	**Mean time to drug preparation[a],**		**Mean time to drug delivery[a], seconds**	
	Diff % (95% CI)	P-value	Diff % (95% CI)	P-value
All drugs	39.5 (22.5-56.5)	<.001	46.6 (27.3-65.8)	<.001
1st drug	8.5 (-15.9-32.8)	.50	16.2 (-10.0-42.4)	.23
2nd drug	28.4 (3.6-53.2)	.03	38.0 (12.7-63.3)	.004
3rd drug	18.8 (5.0-32.6)	.008	25.2 (6.4-44.0)	.009
4th drug	101.9 (68.3-135.6)	<.001	106.9 (70.7-143.1)	<.001

[a] Mean differences were calculated as the values for participants using conventional methods minus the values for those using the mobile app. Mean differences were assessed by using linear models with mixed effects.
1st drug: epinephrine; 2nd drug: midazolam; 3rd drug: 10% dextrose; 4th drug: sodium bicarbonate.
Abbreviation: 95% CI: 95% confidence interval.

© 2021 Seibert JN et al. *JAMA Network Open*.

eTable 6. Subgroup Analysis for Primary and Secondary Outcomes by Years Since Paramedic Certification and EMS Annual Number of Pediatric Interventions

	Paramedic experience			EMS experience	
	< 5 years	5 to 10 years	> 10 years	< 250 pediatric interventions/year	> 250 pediatric interventions/year
**Medication errors, n/N (%)**					
Mobile app	2/104 (1.9%)	7/116 (6.0%)	8/76 (10.5%)	10/164 (6.1%)	7/132 (5.3%)
Conventional method	65/108 (60.2%)	64/104 (61.5%)	62/92 (67.4%)	110/164 (67.1%)	81/140 (57.9%)
OR (95% CI)[a]	263.2 (46.9-934.0)	84.9 (24.5-211.3)	61.0 (16.9-156.5)	79.2 (23.9-262.9)	124.2 (39.1-394.7)
P-value	.28			.51	
**Time to drug preparation, mean (SD)**					
Mobile app	152.8 (57.2)	141.7 (65.0)	144.7 (78.4)	141.6 (55.5)	152.3 (77.2)
Conventional method	176.8 (124.1)	208.0 (113.6)	170.7 (91.8)	181.5 (113.2)	190.5 (111.6)
Mean difference (95% CI)[b]	23.8 (-4.8-52.5)	67.0 (39.0-95.1)	25.7 (-6.4-58.0)	40.4 (17.4-63.4)	38.3 (12.9-63.5)
P-value	.07			.90	
**Time to drug delivery (seconds), mean (SD)**					
Mobile app	190.0 (50.9)	183.9 (65.4)	186.2 (81.7)	182.8 (56.4)	191.4 (74.9)
Conventional method	232.5 (140.5)	250.2 (109.2)	214.6 (92.7)	230 (118.0)	236.8 (117.3)
Mean difference (95% CI)[b]	42.2 (9.7-74.8)	66.6 (34.7-98.6)	28.4 (-8.2-65.1)	47.4 (21.3-73.5)	45.4 (16.7-74.1)
P-value	.29			.92	

[a] logistic regression model with mixed effects and with an interaction term between the level of experience and the intervention.
[b] linear regression model with mixed effects and with an interaction term between the level of experience and the intervention.
Abbreviations: EMS: emergency medical services; n/N: number of errors (n) over number of preparation opportunities (N); OR: odds ratio; 95% CI: 95% confidence interval; SD: standard deviation; TDP: time to drug preparation in seconds; TDD: time to drug delivery in seconds.

© 2021 Seibert JN et al. *JAMA Network Open*.

eReferences.

1. Cheng A, Auerbach M, Hunt EA, et al. Designing and conducting simulation-based research. *Pediatrics*. 2014;133(6):1091-1101. doi:10.1542/peds.2013-3267

2. Bottiger BW, Bernhard M, Knapp J, Nagele P. Influence of EMS-physician presence on survival after out-of-hospital cardiopulmonary resuscitation: systematic review and meta-analysis. *Crit Care*. 2016;20:4. doi:10.1186/s13054-015-1156-6

3. Stenner K, van Even S, Collen A. Early adopters of paramedic prescribing: a qualitative study. *Br Paramed J*. 2019;4(3):57. doi:10.29045/14784726.2019.12.4.3.57

4. Kiguchi T, Okubo M, Nishiyama C, et al. Out-of-hospital cardiac arrest across the world: first report from the International Liaison Committee on Resuscitation (ILCOR). *Resuscitation*. 2020;152:39-49. doi:10.1016/j.resuscitation.2020.02.044

5. Siebert JN, Ehrler F, Combescure C, et al. A mobile device app to reduce time to drug delivery and medication errors during simulated pediatric cardiopulmonary resuscitation: a randomized controlled trial. *J Med Internet Res*. 2017;19(2):e31. doi:10.2196/jmir.7005

6. Siebert JN, Ehrler F, Combescure C, et al. A mobile device application to reduce medication errors and time to drug delivery during simulated paediatric cardiopulmonary resuscitation: a multicentre, randomised, controlled, crossover trial. *Lancet Child Adolesc Health*. 2019;3(5):303-311. doi:10.1016/S2352-4642(19)30003-3

7. de Caen AR, Berg MD, Chameides L, et al. Part 12: Pediatric Advanced Life Support: 2015 American Heart Association guidelines update for cardiopulmonary resuscitation and emergency cardiovascular care. *Circulation*. 2015;132(18 Suppl 2):S526-S542. doi:10.1161/CIR.0000000000000266

8. Merchant RM, Topjian AA, Panchal AR, et al. Part 1: Executive summary: 2020 American Heart Association guidelines for cardiopulmonary resuscitation and emergency

© 2021 Seibert JN et al. *JAMA Network Open*.

cardiovascular care. *Circulation*. 2020;142(16_suppl_2):S337-S357. doi:10.1161/CIR.0000000000000918

9. Huebinger RM, Zaidi HQ, Tataris KL, et al. Retrospective study of midazolam protocol for prehospital behavioral emergencies. *West J Emerg Med*. 2020;21(3):677-683. doi:10.5811/westjem.2020.3.45552

10. Shtull-Leber E, Silbergleit R, Meurer WJ. Pre-hospital midazolam for benzodiazepine-treated seizures before and after the rapid anticonvulsant medication prior to arrival trial: a national observational cohort study. *PLoS One*. 2017;12(3):e0173539. doi:10.1371/journal.pone.0173539

11. Guterman EL, Sanford JK, Betjemann JP, et al. Prehospital midazolam use and outcomes among patients with out-of-hospital status epilepticus. *Neurology*. 2020;doi:10.1212/WNL.0000000000010913

12. Keidan I, Ben-Menachem E, Greenberger S. Safety of extravasated sodium bicarbonate. *Resuscitation*. 2015;97:e7. doi:10.1016/j.resuscitation.2015.04.014

13. Shenoi RP, Timm N, Committee On Drugs, Committee on Pediatric Emergency Medicine. Drugs used to treat pediatric emergencies. *Pediatrics*. 2020;145(1)doi:10.1542/peds.2019-3450

14. Ibanescu G. Acceptance factors and use of information technologies: an empirical study on the use of "Rational Suite" software by the employees of a large IT services company: Quebec University (UQAM), 2011. Accessed June 8, 2021. https://archipel.uqam.ca/3960/1/M11905.pdf

© 2021 Seibert JN et al. *JAMA Network Open*.
