# Supplementary material for: Effect of a Mobile App on Prehospital Medication Errors During Simulated Pediatric Resuscitation: A Randomized Clinical Trial
Source: JAMA Netw Open. 2021 Aug 30;4(8):e2123007. doi: 10.1001/jamanetworkopen.2021.23007 (PMC8406083; doi:10.1001/jamanetworkopen.2021.23007)
Supplement: Supplement 3. — The Pediatric Accurate Medication in Emergency Situations (PedAMINES) Prehospital Group [file jamanetwopen-e2123007-s003.pdf]

Supplemental Online Content: Nonauthor Collaborators

*Indicates required information. Only first name, last name, and suffix will appear in PubMed.

*Group Name(s): Pediatric Accurate Medication in Emergency Situations (PedAMINES) Prehospital Group							
***First Name and Middle Initial(s)**	***Last Name**	***Suffix (eg, Jr, III)**	Academic Degrees	Institution	Location (city, state/province, country)	Role or Contribution, eg, chair, principal investigator	Group (if more than 1 Group listed in the byline) and/or Subgroup (eg, Steering Committee)
Marec	Saillant		EMS-p	Geneva Team Ambulances (GTA)	Geneva, Switzerland	On-site study center coordinator	
Renaud	Grandjean		EMS-p	SK Ambulances	Geneva, Switzerland	On-site study center coordinator	
Annick	Leuenberger		EMS-p	Secours Ambulances Genève (SAG)	Geneva, Switzerland	On-site study center coordinator	
Pascal	Donnet		EMS-p	Service de Sauvetage et de Lutte contre les Incendies Aéroportuaires (SSLIA)	Geneva, Switzerland	On-site study center coordinator	
Philippe	Hauck		EMS-p	Service de Sauvetage et de Lutte contre les Incendies Aéroportuaires (SSLIA)	Geneva, Switzerland	On-site study center coordinator	
Sébastien	Pappalardo		EMS-p	Service d'Incendie et de Secours (SIS)	Geneva, Switzerland	On-site study center coordinator	
Philippe	Nidegger		EMS-p	Ambulance Riviera	La Tour-de-Peilz, Switzerland	On-site study center coordinator	
David	Neel		Flight EMS-p	Air Zermatt SA	Zermatt, Switzerland	On-site study center coordinator	
Stephan	Steinhauser		MD	Höhere Fachschule für Rettungsberufe (HFRB)	Zürich, Switzerland	On-site study center coordinator	
Michel	Ceschi		EMS-p	Servizio Ambulanza Locarnese e Valli (SALVA)	Ticino, Switzerland	On-site study center coordinator	
Bruno	Belli		EMS-p	Servizio Ambulanza Locarnese e Valli (SALVA)	Ticino, Switzerland	On-site study center coordinator	
Sébastien	Ottet		EMS-p	Ambulances du Sud Fribourgeois	Vaulruz, Switzerland	On-site study center coordinator	
Wenceslao	Garcia		MD	Ambulances du Sud Fribourgeois	Vaulruz, Switzerland	On-site study center coordinator	

Supplemental Online Content: Nonauthor Collaborators

*Indicates required information. Only first name, last name, and suffix will appear in PubMed.

***First Name and Middle Initial(s)**	***Last Name**	***Suffix (eg, Jr, III)**	Academic Degrees	Institution	Location (city, state/province, country)	Role or Contribution, eg, chair, principal investigator	Group (if more than 1 Group listed in the byline) and/or Subgroup (eg, Steering Committee)
Yoan	Mollier		EMS-p	Service Communal de la Sécurité (SCS)	Neuchâtel, Switzerland	On-site study center coordinator	
Yves	Vollenweider		EMS-p	Service Communal de la Sécurité (SCS)	Neuchâtel, Switzerland	On-site study center coordinator	
Pierre	Voumard		EMS-p	Service Communal de la Sécurité (SCS)	Neuchâtel, Switzerland	On-site study center coordinator	
Karine	Corbat		EMS-p	Service de Protection et Sauvetage Lausanne (SPSL)	Lausanne, Switzerland	On-site study center coordinator	
Philippe	Robadey		EMS-p	Service de Protection et Sauvetage Lausanne (SPSL)	Lausanne, Switzerland	On-site study center coordinator	
Joël	Bauer		EMS-p	Centre de Secours et d'Urgences (CSU) Morges-Aubonne	Aubonne, Switzerland	On-site study center coordinator	
Cyril	Berger		EMS-p	Centre de Secours et d'Urgences (CSU) Morges-Aubonne	Aubonne, Switzerland	On-site study center coordinator	
