# Supplementary material for: Effect of a Mobile App on Prehospital Medication Errors During Simulated Pediatric Resuscitation: A Randomized Clinical Trial
Source: JAMA Netw Open. 2021 Aug 30;4(8):e2123007. doi: 10.1001/jamanetworkopen.2021.23007 (PMC8406083; doi:10.1001/jamanetworkopen.2021.23007)
Supplement: Supplement 4. — Data Sharing Statement [file jamanetwopen-e2123007-s004.pdf]

Data Sharing Statement

Siebert. Effect of a Mobile App on Prehospital Medication Errors During Simulated Pediatric Resuscitation. *JAMA Netw Open*. Published August 30, 2021. doi:10.1001/jamanetworkopen.2021.23007

Data
Data available: Yes
Data types: Deidentified participant data
How to access data: Johan N. Siebert, MD. Johan.Siebert@hcuge.ch
When available: beginning date: 01-01-2022, end date: 01-01-2027

Supporting Documents
Document types: None

Additional Information
Who can access the data: Data will be made available to qualified external researchers whose proposed use of the data has been approved by their Institutional Review Board.
Types of analyses: Data will be made available for a specified research purpose.
Mechanisms of data availability: Data will be made available upon approval of a proposal and with a signed data access agreement.
Any additional restrictions: The request proposal must include a statistician.
